# Hospital-Level Variations in Rates of Inpatient Urinary Tract Infections in Stroke

**DOI:** 10.3389/fneur.2019.00827

**Published:** 2019-08-06

**Authors:** Michelle Tørnes, David J. McLernon, Max O. Bachmann, Stanley D. Musgrave, Elizabeth A. Warburton, John F. Potter, Phyo Kyaw Myint

**Affiliations:** ^1^Ageing Clinical and Experimental Research Group, School of Medicine, Medical Sciences and Nutrition, Institute of Applied Health Sciences, University of Aberdeen, Aberdeen, United Kingdom; ^2^Medical Statistics Team, School of Medicine, Medical Sciences and Nutrition, Institute of Applied Health Sciences, University of Aberdeen, Aberdeen, United Kingdom; ^3^Norwich Medical School, University of East Anglia, Norwich, United Kingdom; ^4^Addenbrooke's Hospital, Cambridge, United Kingdom; ^5^Stroke Research Group, Norfolk and Norwich University Hospital, Norwich, United Kingdom

**Keywords:** stroke, health services research, acute hospitals, outcome, urinary tract infections

## Abstract

**Background and purpose:** Urinary tract infection (UTI) is one of the most common complications following stroke and has prognostic significance. UTI rates have been shown to vary between hospitals, but it is unclear whether this is due to case-mix differences or heterogeneities in care among hospitals.

**Methods:** A prospective multi-center cohort study of acute stroke patients admitted to eight National Health Service (NHS) acute hospital trusts within the Anglia Stroke & Heart Clinical Network between 2009 and 2011 was conducted. We modeled the association between hospital (as a fixed-effect) and inpatient UTI using a multivariable logistic regression model, adjusting for established patient-level risk factors. We graphically and descriptively analyzed heterogeneities in hospital-level characteristics.

**Results:** We included 2,241 stroke admissions in our analysis; 171 (7.6%) acquired UTI as an inpatient. UTI rates varied significantly between the eight hospitals, ranging from 3 to 11%. The hospital that had the lowest odds of UTI [odds ratio (OR) = 0.50 (95% confidence interval (CI) 0.22–.11)] in adjusted analysis, had the highest number of junior doctors and occupational therapists per five beds of all hospitals. The hospital with the highest adjusted UTI rate [OR=2.69 (1.56–4.64)] was tertiary, the largest and had the highest volume of stroke patients, lowest number of stroke unit beds per 100 admissions, and the highest number of hospital beds per CT scanner.

**Conclusions:** There is hospital-level variation in post-stroke UTI. Our results suggest the potential influence of service characteristics independently of patient-level factors which may be amenable to be addressed to improve the ultimate stroke outcome.

## Introduction

Stroke is the third leading cause of disability in the world, and in 2013 contributed to 113 million disability-adjusted life years (DALYs) (1). Although the initial brain insult and subsequent neurological deficit are a direct consequence of stroke, poor functional outcome is also contributed by post-stroke complications such as urinary tract infections (UTIs) (2–6). UTI is one of the most common complications following stroke, with a reported prevalence of up to 28% (7, 8). It has been estimated that 4.1% of costs associated with length of hospital stay could be saved through the prevention of post-stroke UTI as well as it improving functional outcome (9).

Given its influence on stroke outcomes such as functional recovery, (2–6) length of stay (2, 9–12) and acute care costs, (9) the prevention, early diagnosis, and treatment of UTI is a priority in stroke care. Most research to date has been concerned with identifying patient characteristics that increase the risk of stroke complicated by UTI in order for these patients to be monitored closely for signs of early infection (3, 11, 13–15). Many studies have also tried to determine what processes of care can ensure protection against UTI and have primarily looked at the consequence of catheterization, (13, 16, 17) early mobilization and assessment, (18) and admission to a stroke unit (19). Much less focus has been given to understand whether there is variation in post-stroke UTI during hospital admission between services and if so, what hospital characteristics could lead to higher post-stroke UTI rates.

It has been shown that UTI rates amongst stroke patients vary between different hospitals, in some cases from as low as 5% to as high as 22% (11). Although such variation could be due to differences in case-mix, a study by Tong et al. (12) showed that hospital location is independently associated with odds of acquiring post-stroke UTI. This suggests that factors at the hospital level, such as resource levels and staffing, could be playing a role in UTI incidence during acute hospital stay.

The lack of hospital-level research examining differences in UTI rates following stroke represents an important knowledge gap. Identifying variations in UTI amongst hospitals once case-mix differences are accounted for, and the service-level factors responsible, will highlight opportunities for policy makers and health care planners to reduce post-stroke UTI and, as a result, to influence other outcomes such as length of stay and dependency.

The aim of this study is to investigate whether there are differences in post-stroke UTI rates between hospitals in a United Kingdom (UK) National Health Service (NHS) after accounting for case-mix differences and individual prognostic variables using the Anglia Stroke Clinical Network Evaluation Study (ASCNES). Our secondary objective, if there are unexplained hospital-level variations in post-stroke UTI, is to explore which hospital-level factors may influence post-stroke UTI rates.

## Materials and Methods

### Study Design and Setting

A prospective, multi-center cohort study was conducted at eight acute hospital NHS trusts which participated in ASCNES, which covers the three counties of Suffolk, Norfolk and Cambridgeshire, in the East of England with a catchment population of ~2.5 million. The ASCNES primary outcome of interest was 1-year mortality, whereas inpatient UTI, the focus of this paper, was one of the secondary outcomes of interest. The detailed study protocol has previously been published (20). Ethical approval was obtained from the NRES Committee East of England—Norfolk (REC Reference number 10/H0310/44). Written informed consent for participation was not required for this study in accordance with the national legislation and the institutional requirements.

### Participants

The study population included all patients, aged 18 years or older, admitted to any of the eight hospitals within the Anglia Stroke and Heart Clinical Network (ASHCN) diagnosed with stroke by an accredited stroke physician between October 2009 and September 2011. Stroke was defined as a focal neurological impairment of sudden onset and lasting more than 24 h (or leading to death) as a consequence of an intracerebral ischemic or hemorrhagic event. This definition excludes diagnoses of transient ischemic attacks (TIAs), subdural hematomas, and subarachnoid hemorrhages. Stroke diagnosis was confirmed in all stroke patients through cerebral imaging [either using computed tomography (CT) or magnetic resonance imaging (MRI)]. Diagnoses by the stroke physician were coded using ICD-10. The study sample was systematically selected to include all consecutive stroke patients admitted every third month of this 2-year period, resulting in a total of eight study months. The robustness of this sampling technique has been confirmed (21).

### Participating Hospitals

The participating hospitals, although part of the same network, did not coordinate the care of patients or work together to provide regional care. They were independent NHS Trusts that served their local communities and therefore were individually responsible for managing stroke patients.

### Data Collection

Patient data routinely collected by clinical teams at each participating site for the ASHCN surveys was used in this study. Additional baseline patient and outcome data were retrieved from case records and discharge summaries by the clinical teams. Data was anonymized and sent to the ASCNES coordinating center where it was collated and sent to the research team. Any identifiable patient information was held only at the local NHS trusts and was not accessible to the network and investigators.

### Variables and Data Sources

The primary outcome measure was inpatient UTI recorded during acute hospitalization (yes or no). This variable was collected from the medical notes and discharge summaries of participants and was not routinely collected by ASHCN data collection which formed the basic data of ASCNES. UTI was not explicitly defined in our study and there was no standardized definition of UTI provided to each hospital upon the collection of data. Diagnosis of UTI was at the discretion of the healthcare team treating the patient. The timing of UTI was also not collected.

For our primary objective, the independent variable of interest was hospital ID, coded from 1 to 8. For our secondary objective the independent hospital-level variables of interest were hospital size (number of hospital beds), hospital type (secondary or tertiary), hospital volume of stroke patients (mean number of stroke patients admitted and treated in hospital per month), presence of vascular surgery onsite, distance to neurosurgical facility, number of full-time equivalent (fte) staff per five beds (senior doctors and junior doctors available during weekdays, healthcare associates and nurses, occupational therapists, and physiotherapists), number of total beds present on the stroke unit per 100 stroke admissions, and the total number of hospital beds per CT scanner. The data were collected from clinical leads or service managers at each stroke unit and updated every 6 months over the 2-year study period by research staff (20). For analysis, we took the weighted 2-year average. The denominators used for these hospital-level characteristics (i.e., per 100 stroke admissions) was a means to standardize differences between hospitals by considering the level of resources and staff proportionate to their size or patient volume.

In NHS England, hospitals are either termed secondary or tertiary, dependent on the level of specialist service provided. Tertiary hospitals provide more specialized care in larger, regional or national centers, compared to their secondary counterparts, e.g., neurosurgery units where smaller units are neither viable nor practical. These more centralized hospitals are usually dedicated to providing super-specialty care beyond sub-specialty (e.g., neuro-endocrine surgery is a super speciality of neurosurgery which is a sub-speciality of the speciality of surgery), and therefore have access to more advanced equipment and expertise specific to the conditions in which it subspecializes in. This does not apply to stroke directly, but it is relevant for those who have stroke and require neurosurgical intervention.

Five bed days was used as the denominator as this is how the 2016 national clinical guidelines for stroke reports the recommended staffing levels for UK stroke units, and therefore provides for a comparison (22).

### Statistical Methods

We performed hypothesis testing on important patient-level variables to determine whether they were univariately associated with the presence of UTI. Mann Whitney U test was used to compare differences between groups when the independent variable was continuous and not normally distributed. Chi-squared ($X^{2}$) test was used for categorical, nominal independent variables and the $X^{2}$ test for trend was used for categorical, ordinal independent variables. Included variables were age and sex collected routinely by ACHSN; the presence of stroke risk factors such as atrial fibrillation, previous stroke or TIA, hypertension, diabetes mellitus, hypercholesterolemia, and myocardial infarction or ischemic heart disease collected by clinical teams from case notes; stroke type (ischemic vs. hemorrhagic stroke) collected routinely by ACHSN; Oxfordshire Community Stroke Project classification stroke subtype (total anterior circulation stroke (TACS) vs. other) collected routinely by ACHSN; brain lateralization (i.e., whether stroke symptoms manifested in one hemisphere of the body or was global) collected routinely by ACHSN; pneumonia diagnosis collected from case notes and discharge summaries; pre-stroke modified Rankin Scale score (mRS) collected by clinical teams from case notes; heart rate and body temperature collected by clinical teams from case notes; and whether the patient was admitted to an intensive treatment unit (ITU) collected from the patient administration system.

In order to assess whether post-stroke UTI varies between hospitals, over, and above patient-level prognostic variables, a single-level multivariable logistic regression model was used. Hospital was treated as a fixed effect, with hospital 1 being the reference category, and UTI as the outcome. This approach was decided upon due to the small number of hospitals which would have otherwise provided unreliable hospital effect and variance estimates if a multi-level model was employed (23).

In order to control for confounding variables in the multivariable logistic regression model we selected a number of patient variables a priori, based on a thorough systematic search of the literature. The following established patient-level risk factors were included: age (treated as a continuous variable), sex, the presence of diabetes mellitus, pre-stroke mRS score, pneumonia, and whether the patient was admitted to an ITU. Although stroke severity has been shown to influence post-stroke UTI rates, and we collected data on The National Institutes of Health Stroke Scale (NIHSS) score, we did not include this in our model due to the level of missing data. A total of 73% cases had missing data on NIHSS, and we believe that this variable was only collected in patients that were eligible for thrombolysis. Therefore, we did not multiply impute this variable due to the potential for misclassification bias. Instead, we included whether the patient had a TACS as a proxy as this has previously been shown to correlate well with stroke severity (24–26). Processes of care measures such as catheterization, stroke unit treatment, thrombolysis, early mobilization and timing of assessments, surgical interventions, or scanning were not accounted for in the analyses because we believe them to be intermediate (mediator) variables that lie on the casual pathway between hospital-level factors and stroke patient outcomes (27). Including them in our regression models could otherwise lead to over-adjustment bias (28, 29). For example, if we were to find that low nursing staffing levels were related to higher UTI rates we hypothesize that one explanation for this finding could be that in hospitals with low nursing staffing rates, catheterization rates are increased which predisposes the patient to UTI. If we were to include both variables in the analysis, the process measure (in this case catheterization) would dull/obscure any effect we see between nursing staffing levels and UTI, which could lead to an inaccurate conclusion that nursing levels do not affect UTI rates. To test whether any of the additional patient factors (i.e., that were not selected a priori from our literature review) that were shown to be significantly associated with post-stroke UTI in univariable analysis influenced our findings, we performed a sensitivity analysis.

To fulfill our secondary aim, to explore potential hospital-level predictors of post-stroke UTI, we first provided a descriptive commentary on the service heterogeneities between hospitals. We then used exploratory data analysis which involved plotting the hospital intercept estimates for inpatient UTI from the regression model (estimated adjusted odds ratio of inpatient UTI at each hospital) against the hospital-level characteristics of interest. This method has been recommended by other researchers (23, 30) when the number of higher level units (i.e., hospitals) is too small to allow for the likelihood estimation of hospital effects in multi-level modeling.

To increase power and reduce potential bias of complete case analysis, we performed multiple imputation by chained equations using the MICE package in R (21). All the independent variables of interest, UTI and auxiliary variables (i.e., variables in our dataset that were not used in our model) informed the imputation (Supplementary Table 1). Sixty-four data sets were imputed as the inclusion of auxiliary variables increased the case wise missingness to 64%. Results from the analysis of each dataset were pooled together using Rubin's rules. The distribution of sample characteristics between individuals with complete and incomplete data were compared using the appropriate hypothesis testing. Mann Whitney *U*-test was used to compare differences between groups when the independent variable was continuous and not normally distributed. The $X^{2}$ test was used for categorical, nominal independent variables and the $X^{2}$ test for trend was used for categorical, ordinal independent variables. Complete case analysis was also conducted as a sensitivity analysis so that any differences in results from the multiple imputation analysis could be reported.

Due to limited resources, hospital 2 failed to collect data for the full study period. Patient-level data were only collected in this hospital for October 2009 and January 2010, culminating in a small number of stroke cases for analysis (*n* = 16). To investigate whether this small cluster may affect our results, we performed a sensitivity analysis excluding hospital 2.

Furthermore, data on comorbidities were not collected for 31 cases in hospital 4. To test whether this affects our findings, we performed a further sensitivity analysis excluding these cases.

All analyses were performed using R version 3.3.1 for Windows (22).

### Patient and Public Involvement

The project was managed by the project leader (PKM) who worked in close partnership with the project group of the study and the project steering group. The project steering group included public and patient representatives, recruited through Patient, and Public Involvement in Research (PPIRes). PPIRes members were invited to attend research steering group meetings over the study duration to oversee the project.

## Results

**Figure 1:**
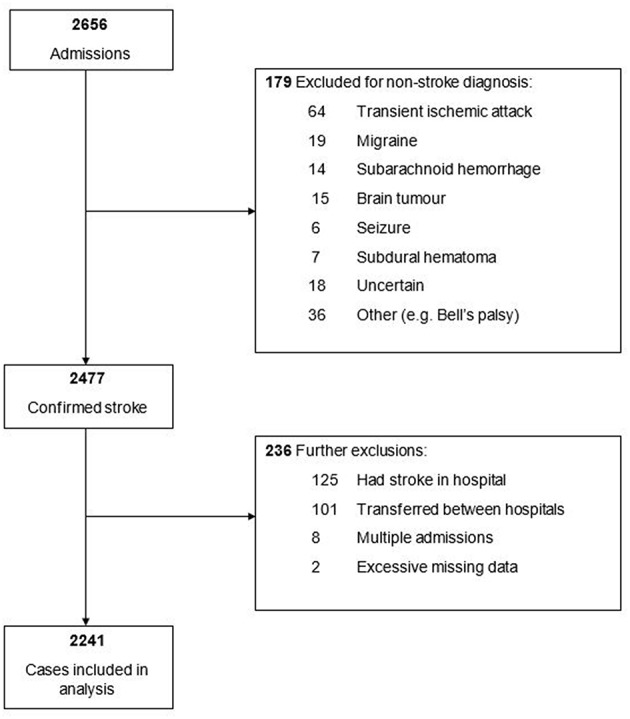
Flow chart for inclusion of patients into study analysis.

Overall, 2,656 patients with suspected stroke were admitted to the eight NHS trusts within the study period and screened for inclusion (see Figure 1). Of these, 2,477 were eventually diagnosed with stroke, with a further 236 excluded for analysis for the following reasons: were in hospital at time of stroke (*n* = 125), transferred between hospitals (both among the eight study hospitals and from or to outside the region) (*n* = 101), multiple admissions (*n* = 8), and excessive missing data (*n* = 2). This left a total of 2,241 stroke patients for the study analysis. The cohort's median age (interquartile range) was 79 (70–86) years, 52% were female and 87% had had an ischemic stroke.

A total of 576 (26%) patients had missing data. Patients had missing data for pre-stroke mRS score (20%), TACS (12%), age (2%), sex (2%), and diabetes mellitus (1%). The reasons for missing data were largely due to hospital 4 failing to collect any data on pre-stroke mRS and failing to collect data on comorbidities for 31 cases. Complete cases and cases with at least one missing variable did not significantly differ with respect to age, sex, presence of diabetes mellitus, pre-stroke mRS score or whether they had a TACS. However, cases with at least one missing variable were more likely to have pneumonia (16 vs. 9%, *P* < 0.001) or be admitted to an ITU (6 vs. 2%, *P* < 0.001) (Supplementary Table 2).

Sample characteristics such as age, sex, stroke risk factors (i.e., atrial fibrillation, diabetes mellitus, hypercholesterolemia), stroke-related factors (i.e., stroke type, subtype, and brain lateralization), pre-stroke independence, pneumonia, ITU admission, heart rate, and body temperature varied between the eight hospitals (Supplementary Table 3).

A total of 171 (7.6%) stroke patients developed inpatient UTI during our study (Table 1). These patients were significantly older (median age 82 vs. 76 years, *P* < 0.001), more likely to be female (68 vs. 51%, *P* < 0.001), more likely to have atrial fibrillation (43 vs. 32%, *P* = 0.004), developed pneumonia (16 vs. 10%, *P* = 0.02), and have had a previous stroke or TIA (40 vs. 30%, *P* = 0.009) compared to those who did not acquire UTI. In addition, these patients were less likely to have hypercholesterolemia (10 vs. 16%, *P* = 0.03) and less likely to be independent (mRS < 3) before admission (72 vs. 81%, *P* = 0.01).

**Table 1:** Characteristics of stroke patients who developed post-stroke UTI and those who did not.

**Patient characteristics**	**UTI**		***p***
	**Yes (*n* = 171)**	**No (*n* = 2,070)**	
**Demographics**			
Age, year [median (IQR)]^*^	82 ± 11	76 ± 13	<0.001
Sex, female	117 (68)	1052 (51)	<0.001
**Stroke risk factors**^**†**^			
Atrial fibrillation	70 (43)	574 (32)	0.004
Previous stroke or TIA	68 (40)	614 (30)	0.009
Hypertensive	118 (69)	1369 (67)	0.60
Diabetes mellitus	36 (21)	336 (16)	0.14
Hypercholesterolemia	17 (10)	338 (16)	0.03
MI/ IHD	36 (21)	482 (24)	0.53
**Stroke-related factors**^**†**^			
Hemorrhagic stroke	19 (11)	256 (13)	0.60
TACS	38 (25)	370 (20)	0.22
No brain lateralization	18 (11)	227 (11)	0.89
Pre-stroke independence (Pre-mRS <3)^‡^	107 (72)	1334 (81)	0.01
Pneumonia^†^	28 (16)	215 (10)	0.02
Heart rate, beats per minute (median, IQR)^*^	80 (70 to 92)	78 (68 to 90)	0.17
Temperature, °C (median, IQR)^*^	36.4 (36 to 36.9)	36.4 (36 to 36.8)	0.29
ITU admission^†^	3 (3)	66 (2)	0.42

Values are number of patients (%) if not indicated.

UTI, urinary tract infection; IQR, interquartile range; TIA, transient ischemic attack; MI/IHD, myocardial infarction or ischemic heart disease; TACS, total anterior circulation stroke; Pre-mRS; pre-stroke modified Rankin scale score; °C, degree Celsius; ITU, intensive treatment unit.

* Mann Whitney U Test.

† $X^{\text{2}}$ test.

‡ $X^{\text{2}}$ test for trend.

Although the overall UTI rate was 7.6%, this varied significantly between different hospitals. The lowest UTI rate was observed in hospital 3 at 3%, whilst the highest was in hospital 5 at 11% (Table 2). In univariable analysis, stroke patients had 1.95- and 2.30-times increased odds of UTI if they were admitted to hospital 7 and 5, respectively instead of to hospital 1. These results did not change greatly after the adjustment for potential confounders, with patients having more than double the odds for developing UTI in hospital 7 [2.35 (95% confidence interval (CI) 1.24 to 4.47)] and hospital 5 [2.69 (1.56–4.64)] compared to hospital 1 (Table 3). In adjusted analysis, patients admitted to hospital 3 were shown to have 50% lower odds of acquiring UTI compared to hospital 1, although this was not statistically significant at the 5% level (*P* = 0.09). Odds of developing UTI did not differ significantly between hospital 1 and hospitals 2, 4, 6, and 8 in unadjusted or adjusted analysis (Table 3).

**Table 2:** Frequency of stroke patients who acquired UTI per hospital, and unadjusted odds ratio (OR) (with 95% CI) for the development of post-stroke UTI according to hospital.

**Hospital**	**N stroke patients admitted during study period**	**Frequency of UTI**		**Unadjusted analysis**	
		***n***	**%**	**OR (95% CI)**	***P***
1	356	19	5	Reference	
2	16	1	6	1.18 (0.15–9.43)	0.87
3	350	10	3	0.52 (0.24–1.14)	0.10
4	144	6	4	0.77 (0.30–1.97)	0.59
5	619	71	11	2.30 (1.36–3.88)	0.002
6	281	24	9	1.66 (0.89–3.09)	0.11
7	252	25	10	1.95 (1.05–3.63)	0.03
8	223	15	7	1.28 (0.64–2.57)	0.49

*UTI, urinary tract infection; OR, odds ratio; CI, confidence interval*.

**Table 3:** Multivariable logistic regression analysis for UTI using multiple imputed dataset (*n* = 2,241).

**Independent variable**	**OR**	**95% CI**	***p***
Age, years	1.03	1.01–1.05	0.003
Sex, female	1.80	1.27–2.56	0.001
ITU admission	0.80	0.24–2.66	0.71
Diabetes mellitus	1.35	0.90–2.03	0.15
Pre-stroke modified rankin score (reference 0)			
1	1.99	1.21–3.26	0.007
2	2.51	1.47–4.30	<0.001
3	2.41	1.42–4.10	0.001
4 & 5	0.85	0.42–1.73	0.66
TACS	1.16	0.78–1.74	0.46
Pneumonia	1.22	0.76–1.95	0.41
Hospital (reference 1)			
2	1.01	0.12–8.43	0.99
3	0.50	0.22–1.11	0.09
4	0.76	0.29–1.97	0.57
5	2.69	1.56–4.64	<0.001
6	1.89	0.98–3.62	0.06
7	2.35	1.24–4.47	0.01
8	1.32	0.64–2.71	0.45

*UTI, urinary tract infection; OR, odds ratio; CI, confidence interval; ITU, intensive treatment unit; TACS, total anterior circulation stroke*.

The results did not differ when using only complete cases for univariable analysis (Supplementary Table 4). However, using only complete cases for multivariable regression differed from the results obtained following multiple imputation. As hospital 4 did not collect any data on pre-stroke mRS score, this was completely excluded from the complete case analysis. Furthermore, the odds ratio of UTI in hospital 3 was reduced from 50 to 35% and became significantly different from hospital 1 in complete case analysis. Although the direction of association did not change, the hospitals with the highest odds of UTI were no longer significantly different from hospital 1 when using complete cases only (Supplementary Table 5).

Patient-level factors that were shown to be significantly associated with post-stroke UTI in multivariable regression analysis were age, sex, and pre-stroke mRS (Table 3). For every 1-year increase in age there was a 3% increase in odds of developing post-stroke UTI. Females had 80% increased odds of developing post-stroke UTI compared to their male counterparts. Patients with pre-stroke mRS scores of 1–3 were shown to have increased odds of 1.99, 2.51, and 2.55, respectively, of post-stroke UTI compared to those patients with a score of 0. No statistically significant difference in UTI rates were observed between patients with a pre-stroke mRS score of 0 and those with a score of 4 or 5. Other patient factors that were not statistically significantly associated with post-stroke UTI were whether the patient was admitted to an ITU, whether the patient had diabetes mellitus, whether the patient had a TACS or whether the patient was diagnosed with pneumonia (Table 3).

Table 4 describes the heterogeneity in service and resource levels between the eight hospitals. Patients admitted to hospital 5 had the greatest odds of developing UTI out of all hospitals, compared to hospital 1. Hospital 5 was the study's largest, had the highest volume of stroke patients, lowest number of stroke unit beds per 100 admissions, highest number of beds per CT scanner and had the second longest distance to neurosurgery (61 miles) with respect to all the other hospitals. Hospital 7, which also had more than double the odds of UTI compared to hospital 1, did not share similar characteristics with hospital 5. This hospital was smaller, secondary, had more stroke unit beds per 100 admissions, less hospital beds per CT scanner and a lower volume of patients with stroke. Compared to all other hospitals, however, this hospital was located furthest away from a vascular surgery site and had the lowest number of junior doctors and occupational therapists per five stroke unit beds. No patterns emerged with respect to any of the other hospital characteristics investigated, including nursing staff levels.

**Table 4:** General characteristics, facilities, services, and stroke unit staffing levels of each hospital included in the study.

**Hospital characteristics**	**1**	**2**	**3**	**4**	**5**	**6**	**7**	**8**
**General characteristics**								
Hospital type	Tertiary	Secondary	Secondary	Secondary	Tertiary	Secondary	Secondary	Secondary
Hospital stroke volume (No. of ASCNES admissions per month)	52	13	46	19	88	57	35	31
**Facilities and services**								
No. of hospital beds	1,000	304	800	500	1,237	611	488	460
No. of stroke unit beds (per 100 admissions)	71	77	54	138	41	55	83	65
No. of hospital beds per CT scanners	500	304	400	250	518	306	244	230
Distance to vascular surgery (miles)	0	18	0	25	0	0	43	30
Distance to neurosurgery (miles)	0	18	58	89	61	38	48	30
Stroke unit staffing levels^*^								
Senior doctors*^†^*	0.34	0.25	0.49	0.47	0.42	0.31	0.62	0.87
Junior doctors*^†^*	0.55	0.65	0.72	0.59	0.56	0.64	0.12	0.25
Health care associates and nurses (band 5–7)	9.2	8	6	7.4	7	5.3	6.5	10
Physiotherapists (band 2–8)	0.55	1	0.79	0.4	0.91	0.78	0.69	1
Occupational therapists (band 3–8)	0.49	0.5	1.4	0.59	0.6	0.58	0.52	1.1

No., number; ASCNES, Anglia Stroke Clinical Network Evaluation Study; CT, computed tomography.

* Number of fte staff per five stroke unit beds (weighted average for the four study periods taken).

† Weekday numbers only.

Furthermore, hospital 3, which had the lowest odds of UTI of all hospitals compared to hospital 1, had the highest number of junior doctors and occupational therapists per five stroke unit beds than any of the other hospitals.

The adjusted odds ratio, compared to hospital 1, of developing UTI for each hospital was plotted against the hospital-level factors of interest listed in Table 4. No discernible patterns were seen for the adjusted odds of UTI in relation to any of the hospital-level characteristics [for example, hospital type, volume of stroke cases, staffing levels (including nursing), etc.] (Supplementary Figures 1–12).

Excluding hospital 2, excluding the 31 cases in hospital 4 that were missing comorbidity data and adding additional comorbidities to our multivariable logistic regression model in separate sensitivity analyses did not affect our findings of hospital-level variation in UTI (see Supplementary Tables 6–8). It must be noted, however, that in our sensitivity analysis including further comorbidities, whilst atrial fibrillation and previous stroke or TIA were not statistically significantly associated with post-stroke UTI, patients with hypercholesterolemia had 50% lower odds of developing UTI after stroke compared to those who did not have hypercholesterolemia [0.50 (0.28–0.86)] (Supplementary Table 7).

## Discussion

### Key Findings

The study has shown that post-stroke UTI rates significantly differ between participating centers with a catchment population of nearly 2.5 million in the East of England, after case-mix differences and individual prognostic variables have been accounted for. This is in keeping with considerable variations we have noted in stroke service provision and staffing across these hospitals (21). Through descriptive analysis, we have also proposed that hospital-level factors such as access to resources and staffing may be partly driving these hospital variations in post-stroke UTI. However, we did not show any clear trends in the graphical exploration between our hospital-level factors of interest and UTI.

### Interpretation of Results

UTI is one of the most common complications following stroke. A 2011 meta-analysis of 87 studies in the acute phase of stroke estimated post-stroke UTI prevalence to be 10%, comparable to our estimate of 7.6% (31). In the present study, the post-stroke UTI rate ranged from as low as 3% to as high as 11% between hospitals. Such variations have been noted in other multi-center studies in acute stroke care (11, 32). Differences in post-stroke UTI incidence rates between studies and sites may be explained in terms of differences in study design, setting, observation periods, stroke population, and patient sampling (15). However, in the present study we have adjusted for important patient-level prognostic variables and have still shown that differences in UTI rates exist between hospitals in an acute care setting. It could therefore be argued that factors at the hospital level play a role in determining UTI following stroke. This clearly has implications for stroke care and suggests to policy makers that hospital factors such as staffing and resources could be optimized to prevent or reduce this complication. Improvements in such resources would then have direct implications for the outcome of patients with stroke given that post-stroke UTI has been predicted to worsen functional recovery, (2–6) lengthen a patient's hospital stay (2, 9–12) and increase acute care costs (9). As far as we are aware, our study is the first to explicitly show variations in UTI rates between hospitals after the adjustment of several patient-level variables in a UK NHS setting.

Only one other study, to our knowledge, has looked at the independent influence of hospital-level factors on UTI rates following stroke. Tong et al. (12) studying 1,000 community hospitals in the United States, showed that urban hospitals were associated with lower odds of UTI compared to rural hospitals, irrespective of the distribution of age, sex and payer status. This may reflect better resourcing and staffing at larger, urban hospitals that are able to treat stroke patients quicker and more effectively, and physiologically monitor patients more closely, thereby avoiding UTI through prevention and risk reduction. In our analysis, instead of investigating hospital location we used hospital type (secondary vs. tertiary) and size as our independent variables of interest. The theoretical basis of hospital type and size playing a role in UTI rates is similar to that suggested for hospital location. That is, larger, tertiary hospitals are likely to be better resourced and staffed compared to their smaller, secondary counterparts, and hence may be able to prevent, monitor and treat UTI in stroke patients more effectively. However, the hospital with the greatest post-stroke UTI rates in our study was both tertiary and the largest. This may be because tertiary centers, which have more specialized equipment and higher expertise, are more likely to deliver thrombectomy (33) whereby patients are often advised to have 24 h bed rest (34). This lack of mobilization could thereby increase the risk of UTI. We further found no discernible trend with UTI rates when exploring this graphically. This appears to be in agreement with a study by Ji et al. (35) that found stroke inpatient complications are not associated with hospital type.

We also noted in our analysis that the hospital with the highest adjusted odds of post-stroke UTI had the highest volume of stroke patients and the lowest number of stroke unit beds per 100 admissions in the study. It could be argued that where volume of stroke patients is high and/ or where there are fewer stroke unit beds, pressure on beds and staff are heightened. This may mean that patients with stroke are at increased risk of being moved out of the stroke unit to a general ward for some of their acute stay. Previous research has shown that stroke unit care is associated with reduced rates of UTI compared to general medical wards because they have more intense, continuous physiological monitoring which allows healthcare professionals to recognize signs of UTI early, and treat it before it becomes symptomatic (36). Ji et al. (35) however, did not find an association between the number of beds and inpatient stroke complications, although it must be noted that this study did not look at UTI specifically.

Access to services may additionally play a role in determining post-stroke UTI rates amongst hospitals. For example, hospital 5, with the highest adjusted UTI rate, had the highest number of beds per CT scanner and had the second longest distance from neurosurgical facilities. Moreover, hospital 7 that also had one of the highest odds of UTI, had the longest distance to vascular surgery facilities. It could be hypothesized that patients admitted to hospitals that have to travel a longer distance to surgical facilities are less likely to be treated as promptly and are at an increased risk of neurological deterioration than those patients admitted to hospitals that have facilities onsite or nearby. This may mean that these patients have a slower recovery and require catheterization due to impaired consciousness and motor function that leads to immobility, dysphasia, and the inability to communicate and transfer to the toilet. This could therefore predispose patients treated in hospitals located a distance away from surgical facilities to be at an increased risk of UTI compared to patients treated in hospitals where facilities are located onsite and treatment can be carried out promptly (37). Similarly, a higher number of hospital beds per CT scanners suggests that there may be a greater demand for CT scanning due to more patients than in hospitals where the bed to CT scanner ratio is smaller. Greater demand for CT scanning in these hospitals may therefore mean that there is a delay in scanning and therefore diagnosis in patients with stroke at these hospitals with higher bed to CT scanner ratios. Again, this could lead to slower treatment and poorer outcomes, which make the patient susceptible to deteriorating and hence at greater risk of developing a UTI.

The hospital in our study that was seen to have the lowest UTI rate was also the hospital with the highest junior doctor and occupational therapy staffing levels per five stroke unit beds. Hospital 7, with more than double the odds of UTI compared to our reference hospital, on the other hand had the lowest number of junior doctors and occupational therapists per five stroke unit beds in the study. However, our study did not appear to show any pattern between post-stroke UTI and nursing levels. These findings are surprising and hard to explain as one would expect that nursing levels would more likely shape post-stroke UTI rates than the number of junior doctors would. Nurses are responsible for the monitoring and general care of stroke patients, and so if staffing levels were limited in this regard one may expect to see an increase in UTI due to, for example, an increased need for catheterization which has been shown to independently increase the odds of UTI development 4-fold (17). In addition, fewer nurses may mean a reduced ability for intensive physiological monitoring, which is important in the early detection and prevention of UTI. It is unclear why occupational therapist staffing may influence UTI control. Staffing levels of physiotherapists may have been easier to explain given that they can mobilize patients earlier, which has been seen to reduce UTI rates in patients with stroke (18). However, it could be surmised that because the role of occupational therapists is to help patients regain independence in the safe functioning of performing daily activities, such as bathing, dressing, transferring to the toilet through training and adaptive measures, higher staffing levels could equate to more time spent with the patient. The higher therapy time with occupational therapists may therefore mean that patients with stroke who are treated in hospitals with higher staffing levels are more able to regain independence in bathing and toileting than those treated where there are fewer occupational therapists per five beds, making them less susceptible to catheterization and unhygienic practices that could lead to UTI development.

The lack of discernible patterns emerging from our graphical exploration of adjusted UTI rates and hospital-level characteristics should not be taken to mean that the above hospital-level factors discussed do not play a role in determining UTI. Instead, what this likely illustrates is the complexity and interplay of many factors at the hospital level that are impacting post-stroke UTI. It also clearly reflects the issues of having a small sample of hospitals to investigate hospital-level characteristics responsible for variation in this outcome.

Furthermore, although we did not set out to explore the patient-level factors influencing post-stroke UTI rates, we demonstrated that age, sex, and pre-stroke mRS score are significantly associated with UTI development. This is in agreement with other studies that have looked at these variables. For example, a 2011 systematic review and meta-analysis of 87 studies concluded that advanced age is independently associated with an increase in UTI in patients with stroke (31). This association may reflect the weaker immune systems and predisposition to multi-comorbidities in elderly patients that make them more susceptible to UTI infections (38, 39). Our study demonstrated that female patients had nearly double the odds of contracting UTI after stroke than their male counterparts. Again, this is in agreement with several other studies, including the study by Reid et al. (40) which showed that, after the adjustment for a number of important covariates, female sex was associated with a 2.06 (1.61–2.68) increased odds in acquiring post-stroke UTI. It has been hypothesized that such an association arises because females are more often fitted with indwelling catheters than males who can be catheterized using condom drainage or peni-flow catheters that carry a smaller risk of UTI development. Additionally, females have shorter urethras which are closer in proximity to the perineum (than in males) where there is therefore greater exposure to infection-causing pathogens that lead to UTIs. Our pre-stroke mRS finding also coincides with a study by Stott et al. who showed that patients with UTI were more likely to be functionally dependent (13).

Although we did not find a significant association between ITU admission, presence of diabetes mellitus, TACS, and pneumonia with post-stroke UTI rates, other studies have indicated they play a role (13, 31, 35, 41–43). The reason for differences in findings may be due to a difference in study design, sample population or how UTI has been diagnosed (i.e., our study did not provide a pre-specified definition).

Finally, in our sensitivity analysis we demonstrated that patients with hypercholesterolemia had 50% lower odds of developing UTI after stroke than those patients who did not have hypercholesterolemia. To our knowledge, this patient factor has not been previously investigated. This inverse association between hypercholesterolemia and UTI development has been seen in other patient groups (not specifically stroke). For example, Iribarren et al. (44) showed that in a 15 year follow up study of patients admitted to hospital, total cholesterol was significantly, inversely related to UTIs (44). It has been argued that this relationship arises from the immuno-protective effect of lipids whereby endotoxins bind rapidly to lipo-proteins and are inactivated (44, 45).

### Strengths and Limitations

The prospective nature of our study is one of its main strengths as it has enabled us to collect a wealth of detailed and accurate data both at the hospital level and at the patient level. This has allowed us to adjust for important individual prognostic variables, giving us assurance that the variations we see in UTI rates between hospitals is not solely related to case-mix differences. The comprehensiveness of the data collected with regards to hospital characteristics is unique to this study. As well as having a large sample size, we also did not restrict our analysis to specific stroke sub-populations (i.e., the elderly, female, certain stroke types), reducing the chance of selection bias and increasing the generalizability of our findings. As our sample included eight NHS hospitals in the East of England that span both urban and rural regions that cover a large catchment population, and because NHS policies are fairly standard, we believe these sites are generally representative of others across the UK. We further reduced the possibility of bias by conducting multiple imputation so as to include patients with missing data in the analyses. Finally, we have assessed hospital-level factors with a robust and underused statistical methodology. This has allowed for a straight-forward visualization of notable patterns in the dataset whilst taking account of the limitations of inference arising from the small group of hospitals sampled.

Our findings should be interpreted in the context of limitations. First, although we prospectively included patients into our study, data regarding UTI diagnosis was collected from discharge summaries. It is possible that patients who died or had more severe strokes were less likely to have UTI coded in their discharge records, which may have led to bias through differential misclassification. However, by including “total number of complications during acute hospital stay” with a specific field for UTI in the guidance notes for data collection, the chances of under-reporting were minimized. Second, we cannot be sure as to whether UTI was acquired prior to admission or during acute hospitalization as we did not record the timing of the event. If UTI was present in these patients at arrival, it would be hard to argue that the hospital-level variance we see in UTI is a result of differences in hospital resources and facilities. Third, we cannot rule out misdiagnosis of UTI as we did not provide a strict definition or check for inter-observer reliability. Fourth, we were restricted with regard to the number of UTI events. This meant that in some hospitals we did not have enough power to detect significant differences in odds of UTI. For example, the non-significant result for hospital 3 in our main analysis could reflect limited power rather than representing no real differences in UTI rates when compared to our reference hospital. This can also explain the lack of significant effect sizes seen in complete cases analysis for the hospitals with the highest odds of UTI. Moreover, we were unable to adjust for established UTI risk factors such as stroke severity. Although data on stroke severity were collected, it was poorly documented and not believed to be missing at random. However, we tried to negate this by including pre-stroke mRS score, whether the patient had a TACS (which has been shown to correlate with stroke severity), (24–26) and ITU admission, along with other established patient risk factors, into the multivariable logistic regression model. Furthermore, as already stated, we were restricted in our analysis of hospital-level predictors of UTI due to the small number of hospitals sampled and were therefore unable to carry out the preferred option of multi-level modeling or uncover any clear relationships. This therefore limits the generalizability of our findings to other healthcare settings outside the UK with differing national policies. Nonetheless, the descriptive comparison of services amongst hospitals is useful in indicating factors that should be studied further, although this does not imply that the other factors in this study that did not appear relevant such as nursing levels should be ignored. Furthermore, our limited number of hospitals did not preclude us from fulfilling our main objective, which was to determine whether post-stroke UTI rates vary between hospitals after the adjustment for case-mix differences. Finally, we cannot fully rule out an alternative hypothesis; that the higher UTI rates we see in some hospitals may reflect better detection of post-stroke infection rather than being a true indicator of poor preventative care.

Future studies should involve a larger number of hospitals so that the hospital-level factors that are driving hospital-level variance in post-stroke UTI can be determined through multi-level modeling. These studies should ensure that data is collected on all the important, established risk factors of inpatient UTI following stroke in order to minimize the potential issues of confounding variables. Additionally, these studies could carry out mediation analysis to assess the process of care measures acting as mediators in the relationship between hospital-level factors and post-stroke UTI. For example, by collecting catheterization data or thrombectomy rates, these variables could be added in a mediation analysis to check whether the relationship between hospital factors (say staffing or hospital type) is attenuated. Furthermore, collecting data on the timing of stroke onset to imaging or access to surgical facilities could test the hypothesis that patients that are treated in hospitals with offsite surgical facilities or which have greater pressure on CT scanners have longer waiting times for treatment/diagnosis, which then increases their susceptibility to UTI due to neurological deterioration. This would provide further insights as to how hospital-level factors play a role in determining outcomes in stroke.

In summary, inpatient UTI is a common complication of acute stroke. Rates of UTI are not only determined by patient-level factors such as age, sex and pre-stroke mRS, but also by heterogeneities in care between hospitals. It appears as though a complex interplay of hospital factors such as patient volume, facility access, staffing levels, and stroke unit capacity likely influences UTI development following stroke.

## Data Availability

The raw data supporting the conclusions of this manuscript will be made available by the authors, without undue reservation, to any qualified researcher.

## Author Contributions

MT provided input into study design, cleaned the data, planned, and undertook the statistical analysis and interpretation of results, reviewed the literature and prepared the abstract and corresponding manuscript. DM and PM supervised the study. DM provided statistical analysis advice and assistance. MB, EW, JP, and PM conceptualized and designed the use of ASCNES and obtained funding. SM was ASCNES study co-ordinator and managed the data. All authors reviewed the abstract and corresponding manuscript and contributed in critical revision and final preparation.

### Conflict of Interest Statement

The authors declare that the research was conducted in the absence of any commercial or financial relationships that could be construed as a potential conflict of interest.
