# Supplementary material for: Hospital-Level Variations in Rates of Inpatient Urinary Tract Infections in Stroke
Source: Front Neurol. 2019 Aug 6;10:827. doi: 10.3389/fneur.2019.00827 (PMC6691802; doi:10.3389/fneur.2019.00827)
Supplement: Supplementary file 1 [file Data_Sheet_1.docx]

Supplementary Material

**CONTENT**

**Tables**

**Supplementary Table 1.** Variables used to inform multiple imputation of missing data

**Supplementary Table 2.** Sample characteristics of complete cases and those with at least one prognostic variable missing (n=2241)

**Supplementary Table 3.** Sample characteristics of the 2241 patients included in analysis per individual hospital

**Supplementary Table 4.** Univariable logistic regression complete case analysis for UTI

**Supplementary Table 5.** Multivariable logistic regression complete case analysis for UTI (n=1665)

**Supplementary Table 6.** Multivariable logistic regression sensitivity analysis for UTI, excluding hospital 2 using multiple imputed dataset (n=2225)

**Supplementary Table 7.** Multivariable logistic regression sensitivity analysis for UTI, including comorbidities that were shown to be associated in univariable analysis, using multiple imputed dataset (n=2241)

**Supplementary Table 8.** Multivariable logistic regression sensitivity analysis for UTI, excluding 31 cases from hospital 4 that did not collect data on comorbidities, using multiple imputed dataset (n=2210)

**Figures**

**Supplementary Figure 1.** Model estimates of UTI odds ratio for each hospital and hospital type. Horizontal line represents an odds ratio of 1 for reference hospital 1; its green colour represents that it is a tertiary hospital. Multivariable regression model was adjusted for age, sex, ITU admission, pre-stroke mRS, diabetes mellitus, TACS and pneumonia after multiple imputation for missing covariate data.

**Supplementary Figure 2.** Model estimates of UTI odds ratio for each hospital against size of hospital (represented as number of total hospital beds). Horizontal line represents an odds ratio of 1 for reference hospital 1. Vertical line represents the size of hospital 1. Multivariable regression model was adjusted for age, sex, ITU admission, pre-stroke mRS, diabetes mellitus, TACS and pneumonia after multiple imputation for missing covariate data.

**Supplementary Figure 3.** Model estimates of UTI odds ratio for each hospital against hospital stroke volume (mean number of stroke patients admitted and treated in each hospital per month). Horizontal line represents an odds ratio of 1 for reference hospital 1. Vertical line represents the stroke volume of hospital 1. Multivariable regression model was adjusted for age, sex, ITU admission, pre-stroke mRS, diabetes mellitus, TACS and pneumonia after multiple imputation for missing covariate data.

**Supplementary Figure 4.** Model estimates of UTI odds ratio for each hospital against presence of vascular surgery onsite. Horizontal line represents an odds ratio of 1 for reference hospital 1; its blue colour represents that it does not have a vascular surgery onsite. Multivariable regression model was adjusted for age, sex, ITU admission, pre-stroke mRS, diabetes mellitus, TACS and pneumonia after multiple imputation for missing covariate data.

**Supplementary Figure 5.** Model estimates of UTI odds ratio for each hospital against miles to neurosurgery. Horizontal line represents an odds ratio of 1 for reference hospital 1. Vertical line represents miles to neurosurgery from reference hospital. Multivariable regression model was adjusted for age, sex, ITU admission, pre-stroke mRS, diabetes mellitus, TACS and pneumonia after multiple imputation for missing covariate data.

**Supplementary Figure 6.** Model estimates of UTI odds ratio for each hospital against number of senior doctors per five stroke unit beds. Horizontal line represents an odds ratio of 1 for reference hospital 1. Vertical line represents the senior doctor staffing level for the reference hospital. Multivariable regression model was adjusted for age, sex, ITU admission, pre-stroke mRS, diabetes mellitus, TACS and pneumonia after multiple imputation for missing covariate data.

**Supplementary Figure 7.** Model estimates of UTI odds ratio for each hospital against number of junior doctors pr five stroke unit beds. Horizontal line represents an odds ratio of 1 for reference hospital 1. Vertical line represents the junior doctor staffing level for our reference hospital. Multivariable regression model was adjusted for age, sex, ITU admission, pre-stroke mRS, diabetes mellitus, TACS and pneumonia after multiple imputation for missing covariate data.

**Supplementary Figure 8.** Model estimates of UTI odds ratio for each hospital against number of nurses per five stroke unit beds. Horizontal line represents an odds ratio of 1 for reference hospital 1. Vertical line represents the nurse staffing levels for our reference hospital. Multivariable regression model was adjusted for age, sex, ITU admission, pre-stroke mRS, diabetes mellitus, TACS and pneumonia after multiple imputation for missing covariate data.

**Supplementary Figure 9.** Model estimates of UTI odds ratio for each hospital against number of occupational therapists per five bed days. Horizontal line represents an odds ratio of 1 for reference hospital 1. Veritical line represents the staffing levels of occupational therapists in our reference hospital. Multivariable regression model was adjusted for age, sex, ITU admission, pre-stroke mRS, diabetes mellitus, TACS and pneumonia after multiple imputation for missing covariate data.

**Supplementary Figure 10.** Model estimates of UTI odds ratio for each hospital against the number of physiotherapists per five stroke unit beds. Horizontal line represents an odds ratio of 1 for reference hospital 1. Vertical line represents the staffing levels of physiotherapists in our reference hospital. Multivariable regression model was adjusted for age, sex, ITU admission, pre-stroke mRS, diabetes mellitus, TACS and pneumonia after multiple imputation for missing covariate data.

**Supplementary Figure 11.** Model estimates of UTI odds ratio for each hospital against number of stroke unit beds per 100 admissions. Horizontal line represents an odds ratio of 1 for reference hospital 1. Verical line represents the number of stroke unit beds for our reference hospital. Multivariable regression model was adjusted for age, sex, ITU admission, pre-stroke mRS, diabetes mellitus, TACS and pneumonia after multiple imputation for missing covariate data.

**Supplementary Figure 12.** Model estimates of UTI odds ratio for each hospital against number of hospital beds per CT scanner. Horizontal line represents an odds ratio of 1 for reference hospital 1. Vertical line represents the number of hospital beds per CT scanner in our reference hospital. Multivariable regression model was adjusted for age, sex, ITU admission, pre-stroke mRS, diabetes mellitus, TACS and pneumonia after multiple imputation for missing covariate data.

**Supplementary Table 1.** Variables used to inform multiple imputation of missing data

| Variable | Measure |
| --- | --- |
| I. Independent Variables |  |
| Trust | 0=Trust 1 1 =Trust 2 2 =Trust 3 3=Trust 4 4=Trust 5 4=Trust 6 5=Trust 7 6=Trust 8 |
| Sex | 0=Male 1=Female |
| Age | Continuous, years |
| Diabetes Mellitus | 0=No 1=Yes |
| Pre-Stroke mRS | 0=0 1=1 2=2 3=3 4=4 & 5 |
| TACS | 0=No 1=Yes |
| Pneumonia | 0=No 1=Yes |
| ITU or HDU admission | 0=No 1=Yes |
| II. Dependent Variable |  |
| UTI | 0=No 1=Yes |
| III. Auxiliary Variables |  |
| Acute hospital LOS | Continuous, days |
| Brain Lateralization | 0=Yes 1=No |
| Discharge mRS | 0=0 1=1 2=2 3=3 4=4 5=5 6=6 |
| Season of Admission | 0=Summer 1=Winter |
| Day of Admission | 0=Weekday 1=Weekend |
| Recurrent Stroke/TIA | 0=No 1=Yes |
| Dementia | 0=No 1=Yes |
| Hypercholesterolemia | 0=No 1=Yes |
| Myocardial Infarction or Ischemic Heart Disease | 0=No 1=Yes |
| Hypertensive | 0=No 1=Yes |
| Previous Cancer | 0=No 1=Yes |
| Active Cancer | 0=No 1=Yes |
| Depression | 0=No 1=Yes |
| Rheumatoid Arthritis | 0=No 1=Yes |
| Chronic Obstructive Pulmonary Disease | 0=No 1=Yes |
| Pre-Stroke Residence | 0=Independent living without formal care  1=Independent living with formal care  2=Institutional care |
| Stroke Type | 0=Ischemic 1=Hemorrhagic |
| Discharge Destination | 0=Independent living without formal care  1=Independent living with formal care  2=Institutional care  3=Interim or rehabilitation setting  4=Death |
| Atrial Fibrillation | 0=No 1=Yes |
| Glucose Concentration on Admission | Continuous, mmol/L |
| Heart Rate | Continuous, beats per minute |
| Temperature | Continuous, ˚C |
| Another Stroke (Complication) | 0=No 1=Yes |
| Seizure (Complication) | 0=No 1=Yes |
| Myocardial Infarction (Complication) | 0=No 1=Yes |
| SU Admission | 0=No 1=Yes |
| Had Thrombolysis | 0=No 1=Yes |

mRS, modified Rankin Scale; TACS, total anterior circulation stroke; ITU, intensive treatment unit; HDU, high dependency unit; UTI; urinary tract infection; LOS, length of stay; TIA, transient ischemic stroke; SU, stroke unit

**Supplementary Table 2.** Sample characteristics of complete cases and those with at least one prognostic variable missing (n=2241)

| Patient Characteristic | Complete Cases  (n=1665) | Cases with at least one missing variable (n=576) | *P* |
| --- | --- | --- | --- |
|  | Median (IQR) or No. (%) | |  |
| Age, y* | 79 (70 to 86) | 79 (70 to 86) | 0.70 |
| Sex, female*†* | 869 (52) | 300 (52) | 1 |
| Diabetes Mellitus*†* | 290 (17) | 82 (15) | 0.22 |
| TACS*†* | 334 (20) | 74 (24) | 0.16 |
| Pre-stroke mRS Score *‡* |  |  | 0.30 |
| 0 | 853 (51) | 61 (48) |  |
| 1 | 314 (19) | 22 (17) |  |
| 2 | 181 (11) | 10 (8) |  |
| 3 | 166 (10) | 19 (15) |  |
| 4 & 5 | 151 (9) | 16 (12) |  |
| Pneumonia*†* | 150 (9) | 93 (16) | <0.001 |
| ITU Admission*†* | 37 (2) | 32 (6) | <0.001 |

IQR, interquartile range; y, year; TACS, total anterior circulation stroke; mRS, modified Rankin Scale; ITU, intensive treatment unit

** Mann Whitney U Test*

*†* $\mathcal{X}$*^2^ test*

*‡* $\mathcal{X}$*^2^ test for trend*

**Supplementary Table 3.** Sample characteristics of the 2241 patients included in analysis per individual hospital

y, year; IQR, interquartile range; TIA, transient ischemic stroke; MI/IHD, myocardial infarction or ischemic heart disease; TACS, total anterior circulation stroke; Pre-mRS, pre-modified Rankin Score; ˚C, degree Celsius; ITU, intensive treatment unit

| Variables | Hospital 1  356  (16) | Hospital 2  16  (1) | Hospital 3  350  (16) | Hospital 4  144  (6) | Hospital 5  619  (28) | Hospital 6  281  (13) | Hospital 7  252  (11) | Hospital 8  223  (10) |
| --- | --- | --- | --- | --- | --- | --- | --- | --- |
| Demographics |  |  |  |  |  |  |  |  |
| Age, y, median (IQR) | 78  (68 to 85) | 87  (81 to 92) | 79  (72 to 86) | 79  (70 to 86) | 79  (71 to 85) | 78  (71 to 85) | 80  (68 to 85) | 80  (71 to 87) |
| Sex, female | 183 (52) | 9 (56) | 197 (56) | 76 (53) | 310 (50) | 115 (55) | 116 (46) | 123 (55) |
| Stroke Risk Factors |  |  |  |  |  |  |  |  |
| Atrial Fibrillation | 63 (27) | 8 (50) | 80 (36) | 32 (26) | 202 (33) | 102 (37) | 91 (37) | 66 (30) |
| Previous Stroke or TIA | 71 (20) | 7 (44) | 97 (28) | 32 (28) | 220 (36) | 92 (33) | 98 (39) | 65 (29) |
| Hypertensive | 229 (64) | 8 (50) | 202 (58) | 56 (39) | 446 (72) | 200 (71) | 187 (74) | 159 (71) |
| Diabetes Mellitus | 49 (14) | 1 (6) | 59 (17) | 17 (12) | 93 (15) | 66 (23) | 44 (17) | 43 (19) |
| Hypercholesterolemia | 48 (13) | 3 (19) | 24 (7) | 7 (6) | 61 (10) | 80 (28) | 38 (15) | 94 (42) |
| MI/IHD | 46 (13) | 3 (19) | 87 (25) | 30 (21) | 142 (23) | 80 (28) | 49 (19) | 81 (36) |
| Stroke-related Factors |  |  |  |  |  |  |  |  |
| Hemorrhagic Stroke | 51 (15) | 0 (0) | 43 (13) | 9 (9) | 74 (12) | 40 (15) | 32 (13) | 26 (12) |
| TACS | 39 (14) | 2 (14) | 99 (30) | 10 (14) | 107 (18) | 57 (21) | 52 (25) | 42 (19) |
| No Brain Lateralization | 51 (15) | 2 (12) | 14 (4) | 9 (9) | 129 (21) | 1 (0) | 30 (12) | 9 (4) |
| Pre-stroke independence (Pre-mRS < 3) | 169 (86) | 9 (56) | 243 (75) | - | 473 (80) | 159 (81) | 213 (87) | 175 (78) |
| Pneumonia | 63 (18) | 1 (6) | 11 (3) | 10 (7) | 73 (12) | 50 (18) | 19 (8) | 16 (7) |
| Heart Rate, beats per minute, median (IQR) | 77  (68 to 88) | 86  (78 to 96) | 78  (68 to 90) | 78  (68 to 90) | 78  (68 to 92) | 78  (68 to 91) | 77  (67 to 89) | 80  (70 to 88) |
| Temperature, ˚C, median (IQR) | 36.5  (36.1 to 37.0) | 36.4  (36.2 to 36.8) | 36.6  (36.3 to 36.9) | 36.5  (36.2 to 37.0) | 36.1  (35.6 to 36.6) | 36.2  (35.8 to 36.6) | 36.5  (36.1 to 36.8) | 36.6  (36.2 to 36.9) |
| ITU Admission | 16 (4) | 0 (0) | 3 (1) | 14 (10) | 15 (2) | 2 (1) | 9 (4) | 10 (4) |

**Supplementary Table 4.** Univariable logistic regression complete case analysis for UTI

| Patient Characteristic | Number of UTI | Proportion of UTI (%) | OR | 95% CI | *Ρ* |
| --- | --- | --- | --- | --- | --- |
| \| Age, y \| β \| 95% CI \| *Ρ* \| \| --- \| --- \| --- \| --- \| \| Age, y \| 1.02 \| 1.02 to 1.02 \| <0.001 \| \| Sex, female \| 1.20 \| 1.10 to 1.31 \| <0.001 \| \| Recurrent Stroke \| 1.17 \| 1.05 to 1.31 \| 0.01 \| \| Diabetes Mellitus \| 1.16 \| 1.03 to 1.31 \| 0.02 \| \| Dementia \| 1.46 \| 1.25 to 1.70 \| <0.001 \| \| Hypercholesterolemia \| 0.85 \| 0.75 to 0.95 \| 0.01 \| \| Hypertensive \| 1.02 \| 0.93 to 1.12 \| 0.66 \| \| Myocardial Infarction or Ischemic Heart Disease* \| 1.07 \| 0.96 to 1.19 \| 0.23 \| \| TIA \| 1.07 \| 0.94 to 1.21 \| 0.30 \| \| Previous Cancer \| 1.23 \| 1.05 to 1.44 \| 0.01 \| \| Active Cancer \| 0.97 \| 0.80 to 1.16 \| 0.72 \| \| Depression \| 1.06 \| 0.86 to 1.29 \| 0.59 \| \| Rheumatoid Arthritis \| 1.10 \| 0.92 to 1.31 \| 0.31 \| \| COPD \| 0.86 \| 0.71 to 1.06 \| 0.15 \| \| Pre-stroke Rankin Score (reference 0) \|  \|  \|  \| \| 1 \| 1.57 \| 1.38 to 1.79 \| <0.001 \| \| 2 \| 1.63 \| 1.39 to 1.91 \| <0.001 \| \| 3 \| 1.94 \| 1.65 to 2.28 \| <0.001 \| \| 4 & 5 \| 1.32 \| 1.13 to 1.55 \| <0.001 \| \| Pre-Stroke Residence (reference Independent living without formal care) \| \| \| \| \| Independent living with formal care \| 1.52 \| 1.31 to 1.77 \| <0.001 \| \| Institution \| 1.13 \| 0.97 to 1.31 \| 0.11 \| \| Hemorrhagic Stroke \| 0.83 \| 0.73 to 0.96 \| 0.01 \| \| Oxford Community Stroke Project Classification (reference LACS) \| \| \| \| \| PACS \| 1.62 \| 1.44 to 1.82 \| <0.001 \| \| POCS \| 1.22 \| 1.05 to 1.42 \| 0.01 \| \| TACS \| 1.66 \| 1.45 to 1.90 \| <0.001 \| \| No Brain Lateralization \| 0.69 \| 0.60 to 0.80 \| <0.001 \| \| Inpatient Complication \| 2.13 \| 1.94 to 2.34 \| <0.001 \| \| Discharge Destination (reference Independent living without formal care) \| \| \| \| \| Independent living with formal care \| 2.56 \| 2.24 to 2.93 \| <0.001 \| \| Institution \| 4.44 \| 3.91 to 5.05 \| <0.001 \| \| Interim/Rehab Setting \| 2.61 \| 2.31 to 2.94 \| <0.001 \| \| Death \| 1.15 \| 1.03 to 1.28 \| 0.01 \| \| Winter Admission \| 1.20 \| 1.09 to 1.31 \| <0.001 \| \| Weekend Admission \| 1.08 \| 0.98 to 1.20 \| 0.12 \| \| Stroke Unit (reference Unit 1) \|  \|  \|  \| \| 2 \| 2.69 \| 1.58 to 4.58 \| <0.001 \| \| 3 \| 1.19 \| 1.02 to 1.39 \| 0.03 \| \| 4 \| 1.24 \| 1.01 to 1.53 \| 0.04 \| \| 5 \| 0.86 \| 0.75 to 0.99 \| 0.03 \| \| 6 \| 1.11 \| 0.94 to 1.31 \| 0.22 \| \| 7 \| 1.18 \| 1.00 to 1.41 \| 0.05 \| \| 8 \| 0.86 \| 0.72 to 1.03 \| 0.11 \|   Age, y | 171 | 8 | 1.04 | 1.02 to 1.06 | <0.001 |
| Sex, female | 117 v. 54 | 10 v. 5 | 2.09 | 1.51 to 2.94 | <0.001 |
| ITU Admission | 3 v. 168 | 4 v. 8 | 0.54 | 0.13 to 1.48 | 0.30 |
| Diabetes Mellitus | 36 v. 134 | 10 v. 7 | 1.36 | 0.91 to 1.98 | 0.12 |
| Pre-stroke mRS (reference 0) |  |  |  |  | <0.001 |
| 1 | 35 v. 46 | 10 v. 5 | 2.19 | 1.38 to 3.46 | <0.001 |
| 2 | 26 v. 46 | 14 v. 5 | 2.97 | 1.77 to 4.91 | <0.001 |
| 3 | 30 v. 46 | 16 v. 5 | 3.65 | 2.22 to 5.94 | <0.001 |
| 4 & 5 | 11 v. 46 | 7 v. 5 | 1.33 | 0.64 to 2.53 | 0.41 |
| TACS | 38 v. 115 | 9 v. 7 | 1.30 | 0.87 to 1.89 | 0.18 |
| Pneumonia | 28 v. 143 | 12 v. 7 | 1.69 | 1.08 to 2.56 | 0.02 |
| Hospital (reference 1) |  |  |  |  | <0.001 |
| 2 | 1 v. 19 | 6 v. 5 | 1.18 | 0.06 to 6.33 | 0.87 |
| 3 | 10 v. 19 | 3 v. 5 | 0.52 | 0.23 to 1.11 | 0.10 |
| 4 | 6 v. 19 | 4 v. 5 | 0.77 | 0.27 to 1.87 | 0.59 |
| 5 | 71 v. 19 | 11 v. 5 | 2.30 | 1.39 to 3.98 | 0.002 |
| 6 | 24 v. 19 | 9 v. 5 | 1.66 | 0.89 to 3.12 | 0.11 |
| 7 | 25 v. 19 | 10 v. 5 | 1.95 | 1.05 to 3.67 | 0.03 |
| 8 | 15 v. 19 | 7 v. 5 | 1.28 | 0.63 to 2.57 | 0.49 |

UTI, urinary tract infection; OR, odds ratio; CI, confidence interval; y, year; ITU, intensive treatment unit; mRS, modified Rankin Scale; TACS, total anterior circulation stroke

**Supplementary Table 5.** Multivariable logistic regression complete case analysis for UTI (n=1665)

| Patient Characteristic | Number of UTI | Proportion of UTI (%) | OR | 95% CI | *Ρ* |
| --- | --- | --- | --- | --- | --- |
| Age, y | 137 | 8 | 1.04 | 1.02 to 1.06 | <0.001 |
| Sex, female | 99 v. 38 | 11 v. 5 | 2.14 | 1.44 to 3.24 | <0.001 |
| ITU admission | 2 v. 135 | 5 v. 8 | 0.83 | 0.13 to 2.94 | 0.80 |
| Diabetes Mellitus | 28 v. 109 | 10 v. 8 | 1.24 | 0.77 to 1.94 | 0.37 |
| Pre-stroke mRS (reference 0) |  |  |  |  | 0.01 |
| 1 | 33 v. 44 | 1.1 v. 0.5 | 2.01 | 1.21 to 3.31 | 0.006 |
| 2 | 25 v. 44 | 1.4 v. 0.5 | 2.50 | 1.42 to 4.35 | 0.001 |
| 3 | 25 v. 44 | 1.5 v. 0.5 | 2.16 | 1.21 to 3.80 | 0.008 |
| 4 & 5 | 10 v. 44 | 0.7 v. 0.5 | 0.82 | 0.37 to 1.70 | 0.62 |
| TACS | 38 v. 115 | 9 v. 7 | 1.22 | 0.77 to 1.88 | 0.39 |
| Pneumonia | 20 v. 117 | 13 v. 8 | 1.07 | 0.60 to 1.83 | 0.82 |
| Hospital  (reference 1) |  |  |  |  | <0.001 |
| 2 | 1 v. 11 | 7 v. 7 | 0.87 | 0.04 to 5.43 | 0.90 |
| 3 | 9 v. 11 | 3 v. 7 | 0.35 | 0.14 to 0.89 | 0.03 |
| 4 | - | - | - | - | - |
| 5 | 65 v. 11 | 11 v. 7 | 1.96 | 1.01 to 4.10 | 0.06 |
| 6 | 18 v. 11 | 9 v. 7 | 1.64 | 0.73 to 3.83 | 0.23 |
| 7 | 19 v. 11 | 9 v.7 | 1.64 | 0.74 to 3.78 | 0.23 |
| 8 | 14 v. 11 | 6 v. 7 | 0.91 | 0.39 to 2.15 | 0.82 |

UTI; urinary tract infection; OR, odds ratio; CI, confidence interval; y, year; ITU, intensive treatment unit; mRS, modified Rankin Scale; TACS, total anterior circulation stroke

**Supplementary Table 6.** Multivariable logistic regression sensitivity analysis for UTI, excluding hospital 2 using multiple imputed dataset (n=2225)

| Patient Characteristic | OR | 95% CI | *Ρ* |
| --- | --- | --- | --- |
| Age, y | 1.03 | 1.01 to 1.05 | 0.003* |
| Sex, female | 1.78 | 1.25 to 2.53 | 0.001* |
| ITU Admission | 0.81 | 0.24 to 2.69 | 0.73 |
| Diabetes Mellitus | 1.34 | 0.89 to 2.01 | 0.16 |
| Pre-stroke mRS (reference 0) |  |  | <0.001* |
| 1 | 2.04 | 1.26 to 3.30 | 0.004* |
| 2 | 2.62 | 1.53 to 4.48 | <0.001* |
| 3 | 2.55 | 1.49 to 4.34 | <0.001* |
| 4 & 5 | 0.90 | 0.44 to 1.83 | 0.77 |
| TACS | 1.18 | 0.78 to 1.77 | 0.44 |
| Pneumonia | 1.22 | 0.76 to 1.95 | 0.41 |
| Hospital (reference 1) |  |  |  |
| 3 | 0.50 | 0.22 to 1.11 | 0.09 |
| 4 | 0.76 | 0.29 to 1.97 | 0.57 |
| 5 | 2.71 | 1.57 to 4.67 | <0.001* |
| 6 | 1.90 | 0.99 to 3.64 | 0.05 |
| 7 | 2.36 | 1.24 to 4.50 | 0.01* |
| 8 | 1.32 | 0.64 to 2.72 | 0.44 |

UTI; urinary tract infection; OR, odds ratio; CI, confidence interval; y, year; ITU, intensive treatment unit; mRS, modified Rankin Scale; TACS, total anterior circulation stroke

**Supplementary Table 7.** Multivariable logistic regression sensitivity analysis for UTI, including comorbidities that were shown to be associated in univariable analysis, using multiple imputed dataset (n=2241)

| Patient Characteristic | OR | 95% CI | *Ρ* |
| --- | --- | --- | --- |
| Age, y | 1.02 | 1.01 to 1.04 | 0.001 |
| Sex, female | 1.82 | 1.28 to 2.59 | 0.01 |
| ITU Admission | 0.75 | 0.22 to 2.54 | 0.65 |
| Diabetes Mellitus | 1.43 | 0.95 to 2.16 | 0.09 |
| Atrial Fibrillation | 1.28 | 0.90 to 1.82 | 0.16 |
| Previous Stroke or TIA | 1.26 | 0.89 to 1.79 | 0.19 |
| Hypercholesterolemia | 0.50 | 0.28 to 0.86 | 0.01 |
| Pre-stroke mRS (reference 0) |  |  |  |
| 1 | 1.93 | 1.17 to 3.20 | 0.01 |
| 2 | 2.37 | 1.37 to 4.11 | 0.002 |
| 3 | 2.25 | 1.31 to 3.86 | 0.003 |
| 4 & 5 | 0.77 | 0.38 to 1.57 | 0.47 |
| TACS | 1.17 | 0.78 to 1.76 | 0.44 |
| Pneumonia | 1.19 | 0.74 to 1.91 | 0.47 |
| Hospital (reference 1) |  |  |  |
| 2 | 0.98 | 0.12 to 8.29 | 0.99 |
| 3 | 0.46 | 0.21 to 1.03 | 0.06 |
| 4 | 0.73 | 0.28 to 1.89 | 0.51 |
| 5 | 2.50 | 1.45 to 4.33 | 0.001 |
| 6 | 1.95 | 1.01 to 3.76 | 0.05 |
| 7 | 2.20 | 1.15 to 4.20 | 0.02 |
| 8 | 1.55 | 0.74 to 3.23 | 0.24 |

UTI; urinary tract infection; OR, odds ratio; CI, confidence interval; y, year; ITU, intensive treatment unit; mRS, modified Rankin Scale; TACS, total anterior circulation stroke

**Supplementary Table 8.** Multivariable logistic regression sensitivity analysis for UTI, excluding 31 cases from hospital 4 that did not collect data on comorbidities, using multiple imputed dataset (n=2210)

UTI, urinary tract infection; OR, odds ratio; CI, confidence interval; y, year; ITU, intensive treatment unit; mRS, modified Rankin Scale; TACS, total anterior circulation stroke

| Patient Characteristic | OR | 95% CI | *Ρ* |
| --- | --- | --- | --- |
| Age, y | 1.03 | 1.01 to 1.05 | 0.002 |
| Sex, female | 1.83 | 1.29 to 2.61 | 0.001 |
| ITU Admission | 0.80 | 0.24 to 2.69 | 0.72 |
| Diabetes Mellitus | 1.35 | 0.90 to 2.02 | 0.15 |
| Pre-stroke mRS (reference 0) |  |  |  |
| 1 | 2.00 | 1.23 to 3.25 | 0.005 |
| 2 | 2.51 | 1.45 to 4.35 | 0.001 |
| 3 | 2.41 | 1.42 to 4.11 | 0.001 |
| 4 & 5 | 0.85 | 0.42 to 1.73 | 0.65 |
| TACS | 1.15 | 0.76 to 1.74 | 0.51 |
| Pneumonia | 1.15 | 0.71 to 1.85 | 0.57 |
| Hospital (reference 1) |  |  |  |
| 2 | 1.00 | 0.12 to 8.30 | 1.00 |
| 3 | 0.49 | 0.22 to 1.10 | 0.08 |
| 4 | 0.78 | 0.28 to 2.19 | 0.64 |
| 5 | 2.69 | 1.56 to 4.64 | <0.001 |
| 6 | 1.91 | 0.99 to 3.67 | 0.05 |
| 7 | 2.35 | 1.23 to 4.47 | 0.01 |
| 8 | 1.31 | 0.64 to 2.69 | 0.46 |


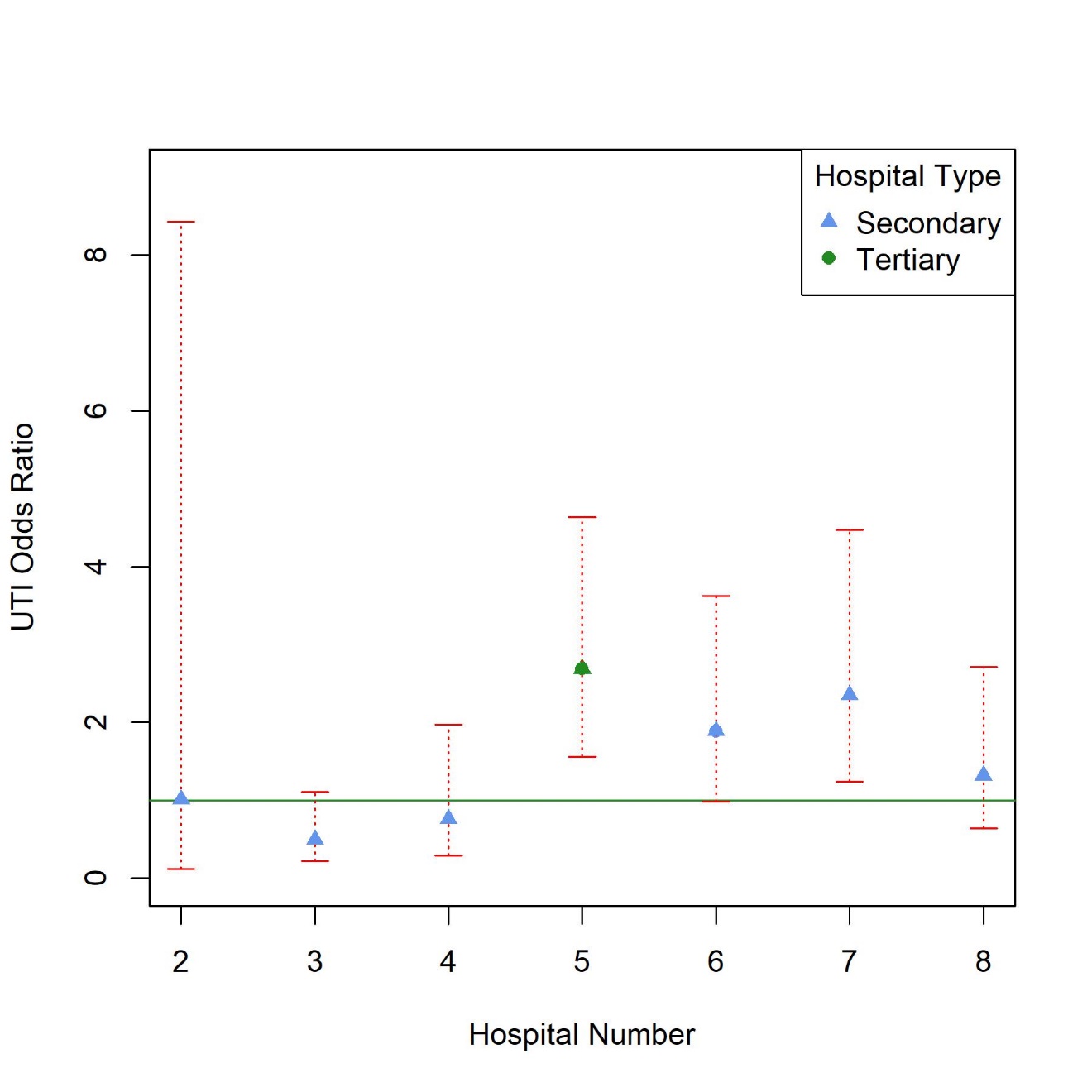


**Supplementary Figure 1.** Model estimates of UTI odds ratio for each hospital and hospital type. Horizontal line represents an odds ratio of 1 for reference hospital 1; its green colour represents that it is a tertiary hospital. Multivariable regression model was adjusted for age, sex, ITU admission, pre-stroke mRS, diabetes mellitus, TACS and pneumonia after multiple imputation for missing covariate data.


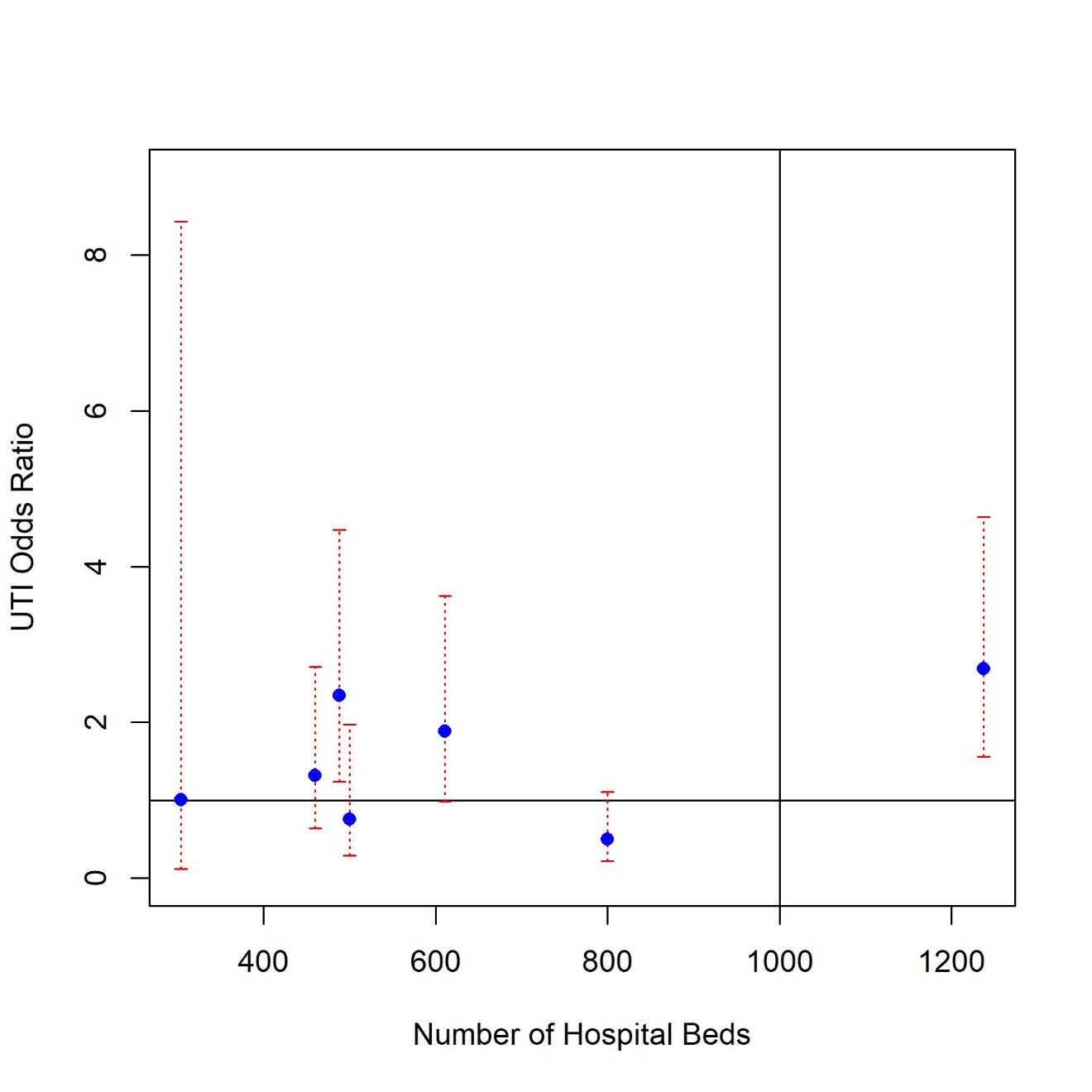


**Supplementary Figure 2.** Model estimates of UTI odds ratio for each hospital against size of hospital (represented as number of total hospital beds). Horizontal line represents an odds ratio of 1 for reference hospital 1. Vertical line represents the size of hospital 1. Multivariable regression model was adjusted for age, sex, ITU admission, pre-stroke mRS, diabetes mellitus, TACS and pneumonia after multiple imputation for missing covariate data.


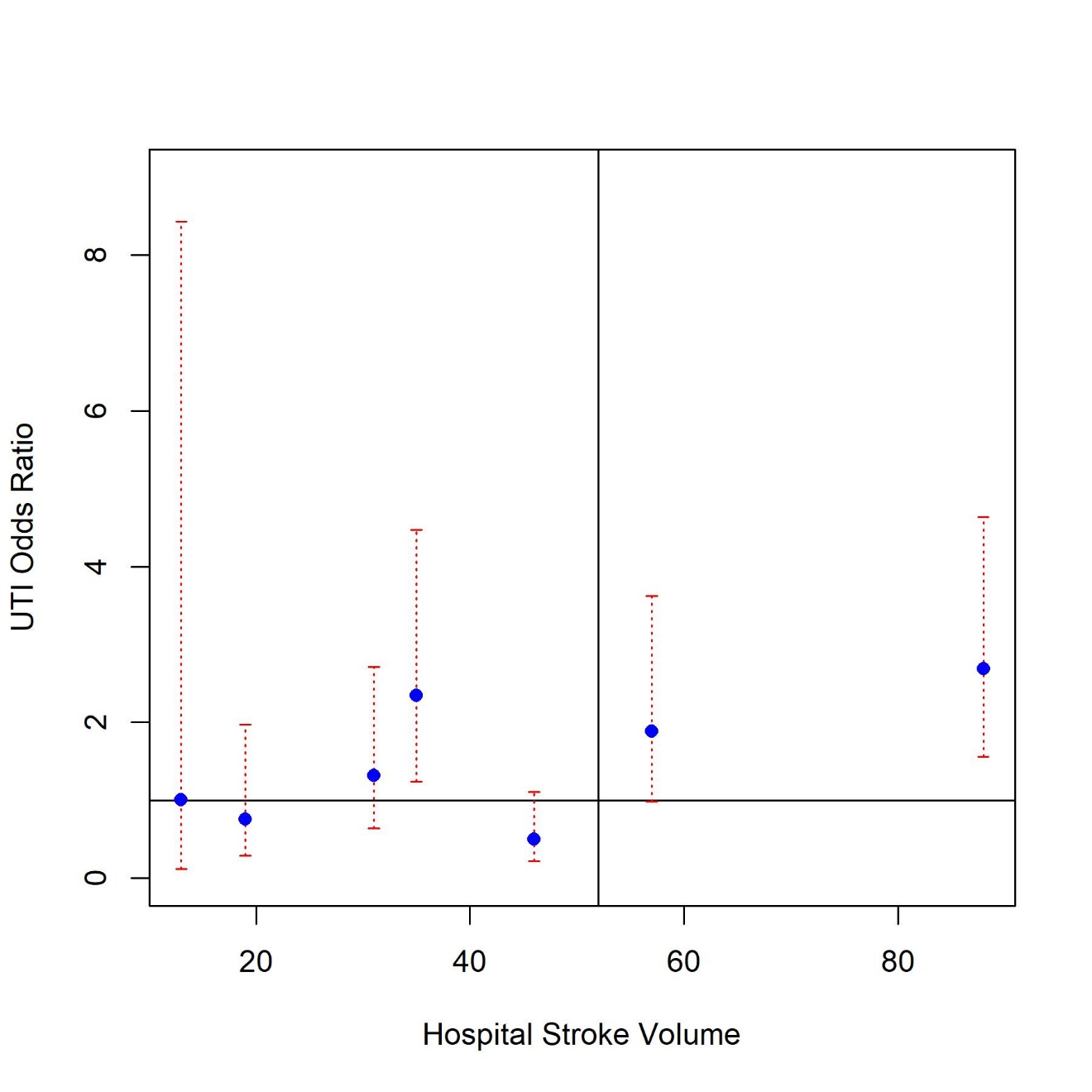


**Supplementary Figure 3.** Model estimates of UTI odds ratio for each hospital against hospital stroke volume (mean number of stroke patients admitted and treated in each hospital per month). Horizontal line represents an odds ratio of 1 for reference hospital 1. Vertical line represents the stroke volume of hospital 1. Multivariable regression model was adjusted for age, sex, ITU admission, pre-stroke mRS, diabetes mellitus, TACS and pneumonia after multiple imputation for missing covariate data.


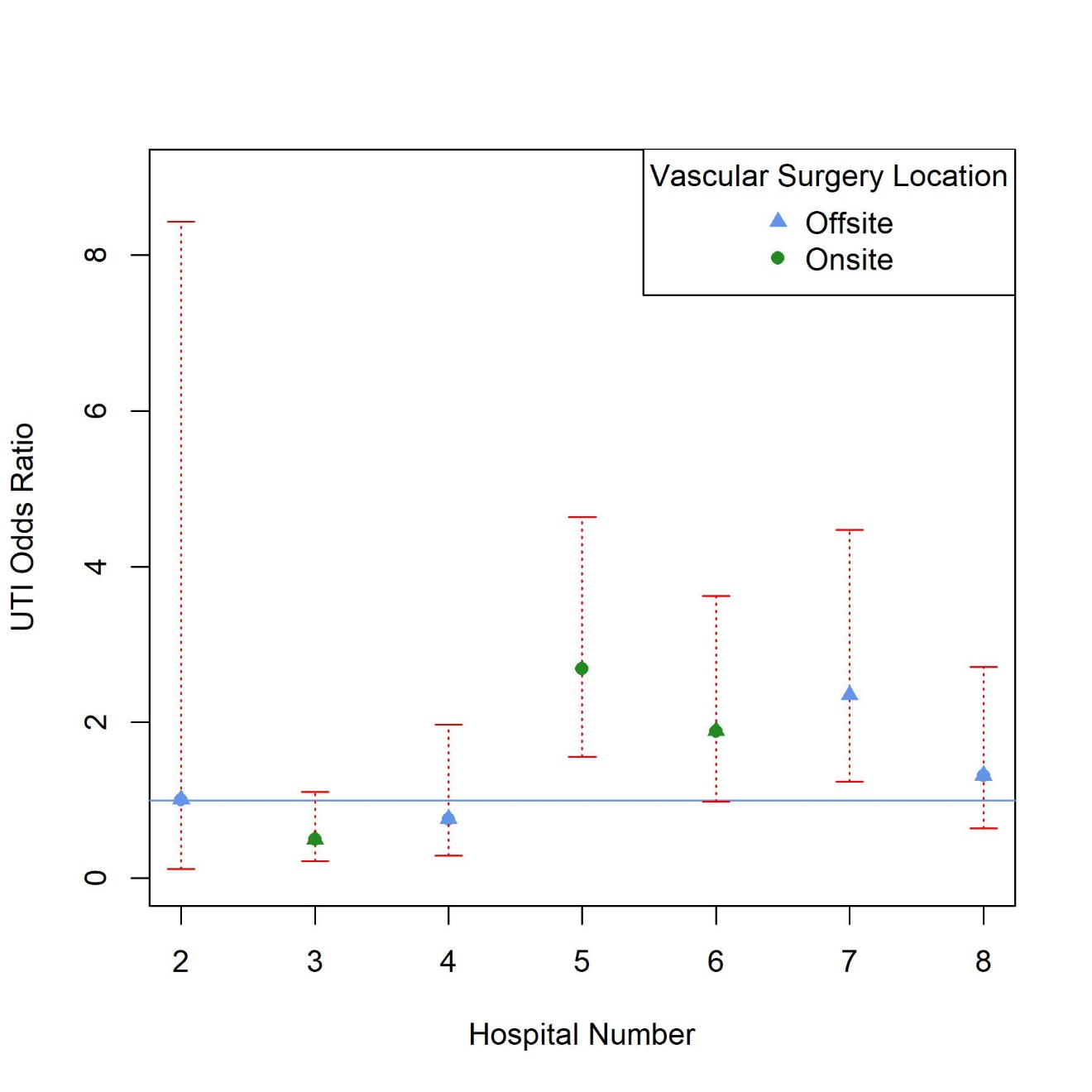


**Supplementary Figure 4.** Model estimates of UTI odds ratio for each hospital against presence of vascular surgery onsite. Horizontal line represents an odds ratio of 1 for reference hospital 1; its blue colour represents that it does not have a vascular surgery onsite. Multivariable regression model was adjusted for age, sex, ITU admission, pre-stroke mRS, diabetes mellitus, TACS and pneumonia after multiple imputation for missing covariate data.


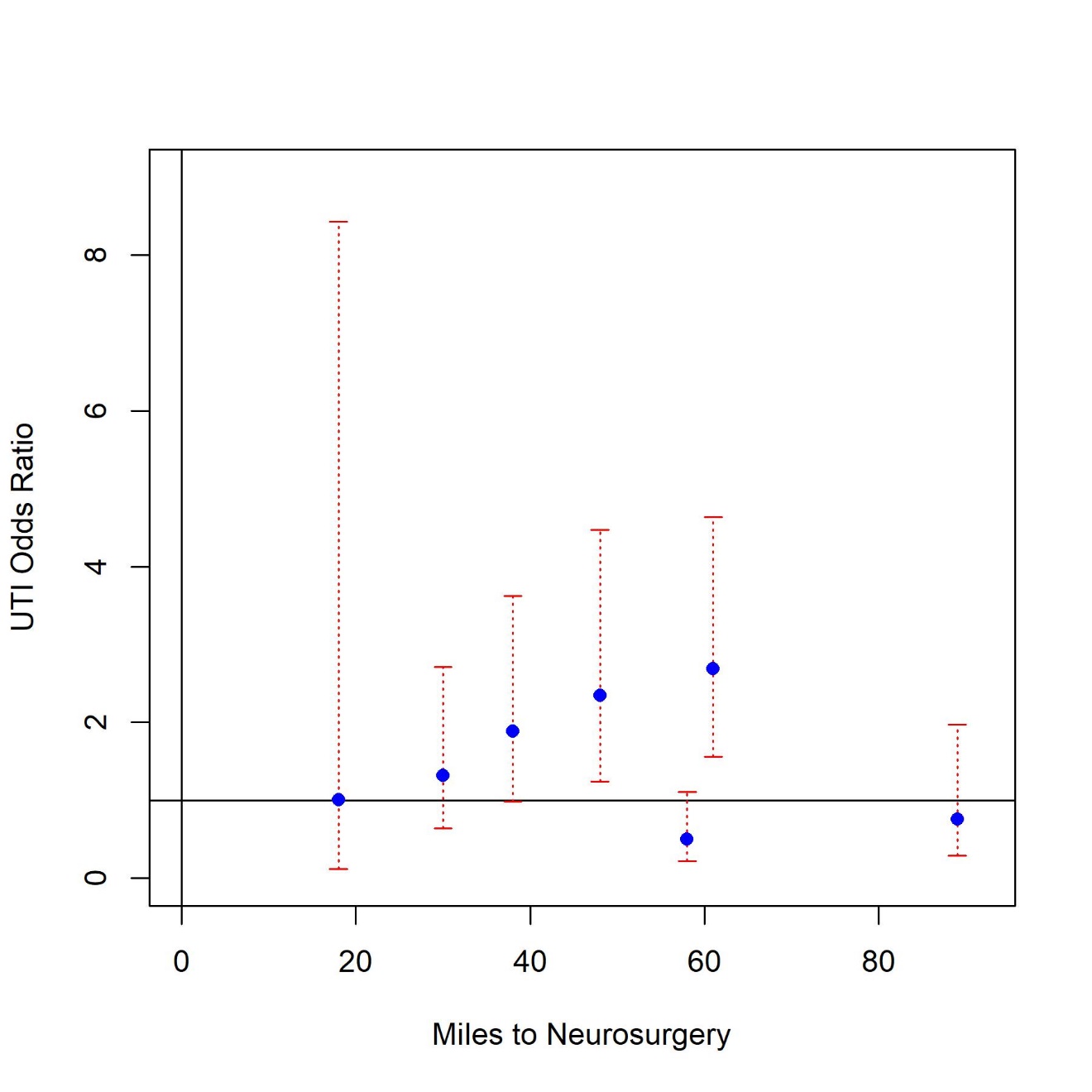


**Supplementary Figure 5.** Model estimates of UTI odds ratio for each hospital against miles to neurosurgery. Horizontal line represents an odds ratio of 1 for reference hospital 1. Vertical line represents miles to neurosurgery from reference hospital. Multivariable regression model was adjusted for age, sex, ITU admission, pre-stroke mRS, diabetes mellitus, TACS and pneumonia after multiple imputation for missing covariate data.


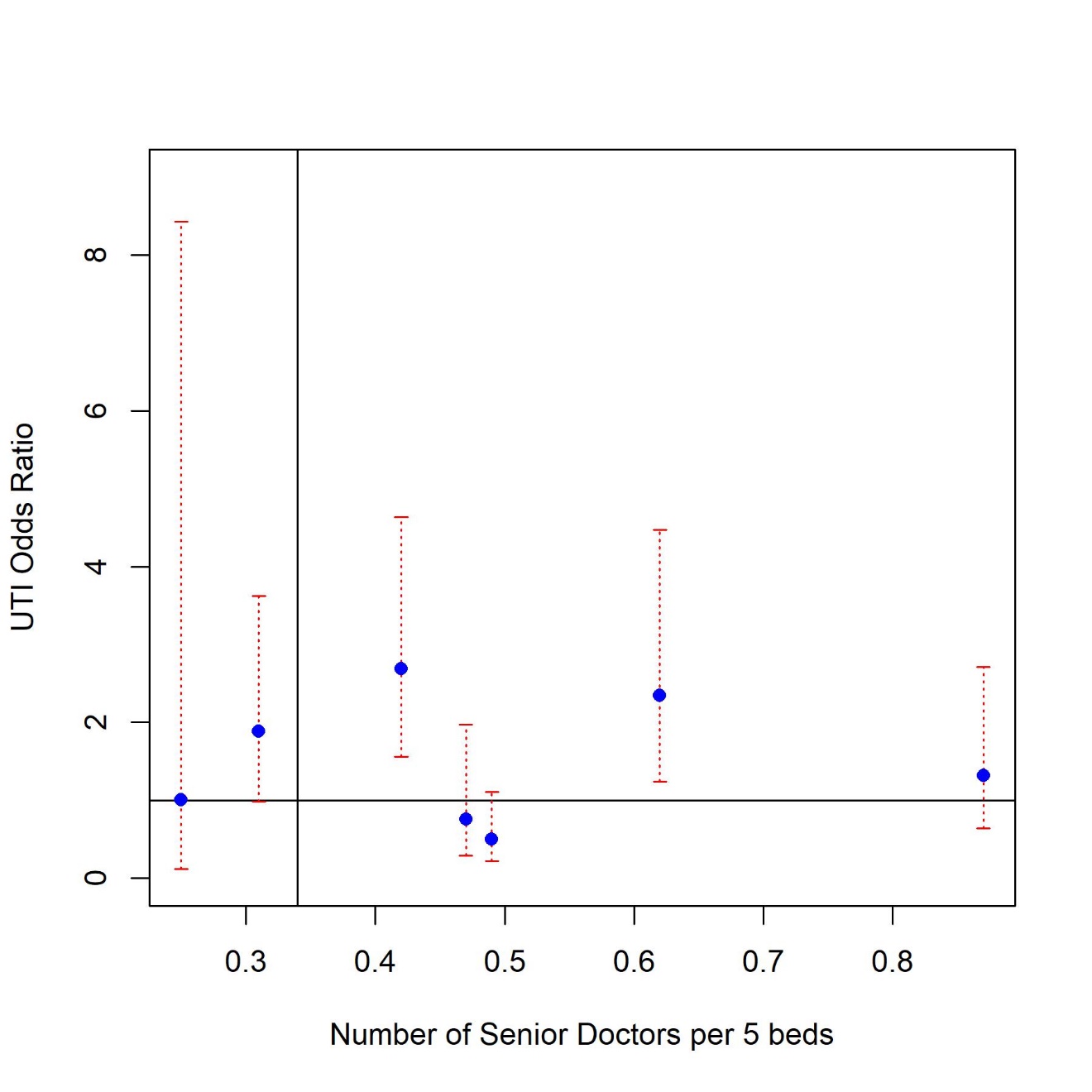


**Supplementary Figure 6.** Model estimates of UTI odds ratio for each hospital against number of senior doctors per five stroke unit beds. Horizontal line represents an odds ratio of 1 for reference hospital 1. Vertical line represents the senior doctor staffing level for the reference hospital. Multivariable regression model was adjusted for age, sex, ITU admission, pre-stroke mRS, diabetes mellitus, TACS and pneumonia after multiple imputation for missing covariate data.


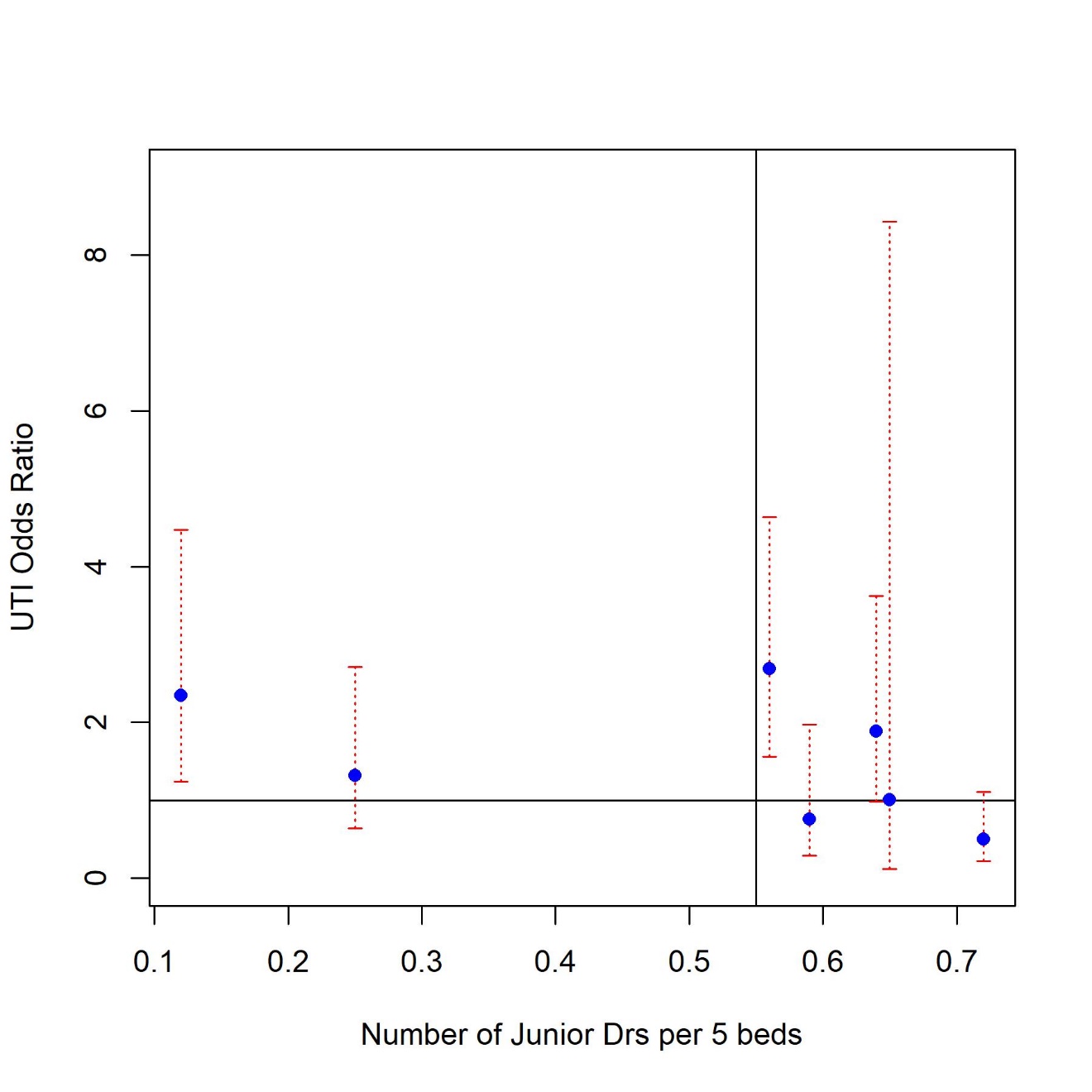


**Supplementary Figure 7.** Model estimates of UTI odds ratio for each hospital against number of junior doctors pr five stroke unit beds. Horizontal line represents an odds ratio of 1 for reference hospital 1. Vertical line represents the junior doctor staffing level for our reference hospital. Multivariable regression model was adjusted for age, sex, ITU admission, pre-stroke mRS, diabetes mellitus, TACS and pneumonia after multiple imputation for missing covariate data.


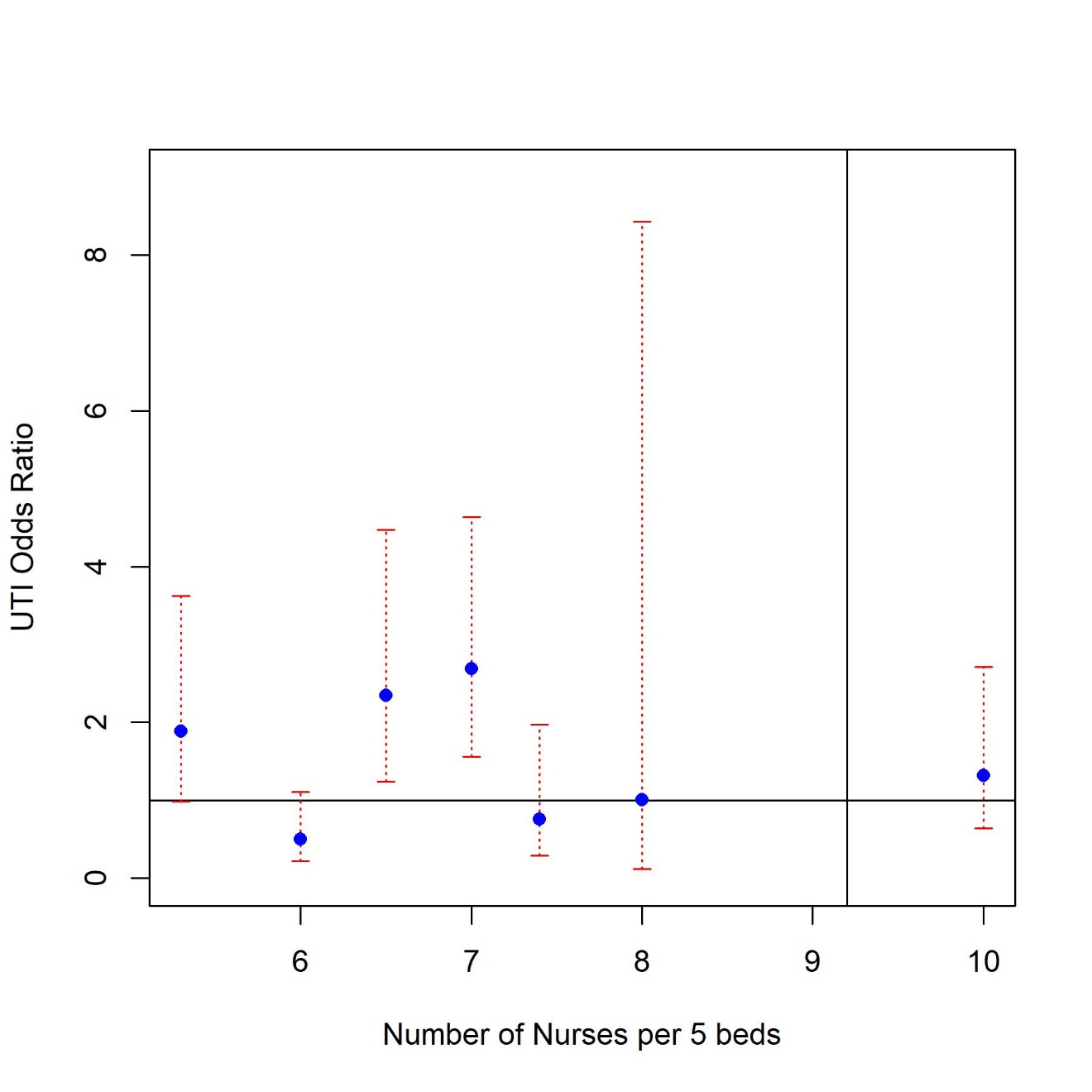


**Supplementary Figure 8.** Model estimates of UTI odds ratio for each hospital against number of nurses per five stroke unit beds. Horizontal line represents an odds ratio of 1 for reference hospital 1. Vertical line represents the nurse staffing levels for our reference hospital. Multivariable regression model was adjusted for age, sex, ITU admission, pre-stroke mRS, diabetes mellitus, TACS and pneumonia after multiple imputation for missing covariate data.


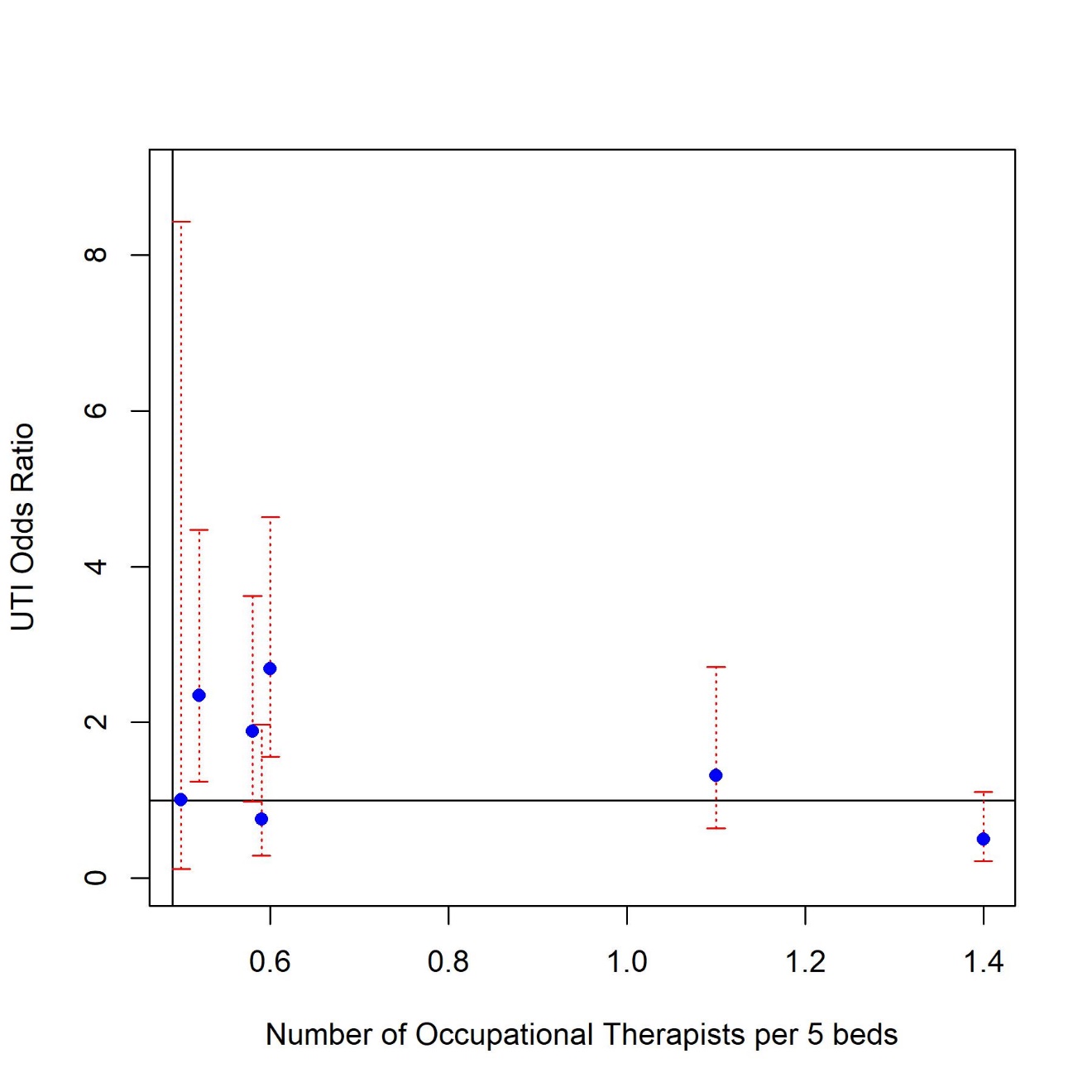


**Supplementary Figure 9.** Model estimates of UTI odds ratio for each hospital against number of occupational therapists per five bed days. Horizontal line represents an odds ratio of 1 for reference hospital 1. Veritical line represents the staffing levels of occupational therapists in our reference hospital. Multivariable regression model was adjusted for age, sex, ITU admission, pre-stroke mRS, diabetes mellitus, TACS and pneumonia after multiple imputation for missing covariate data.


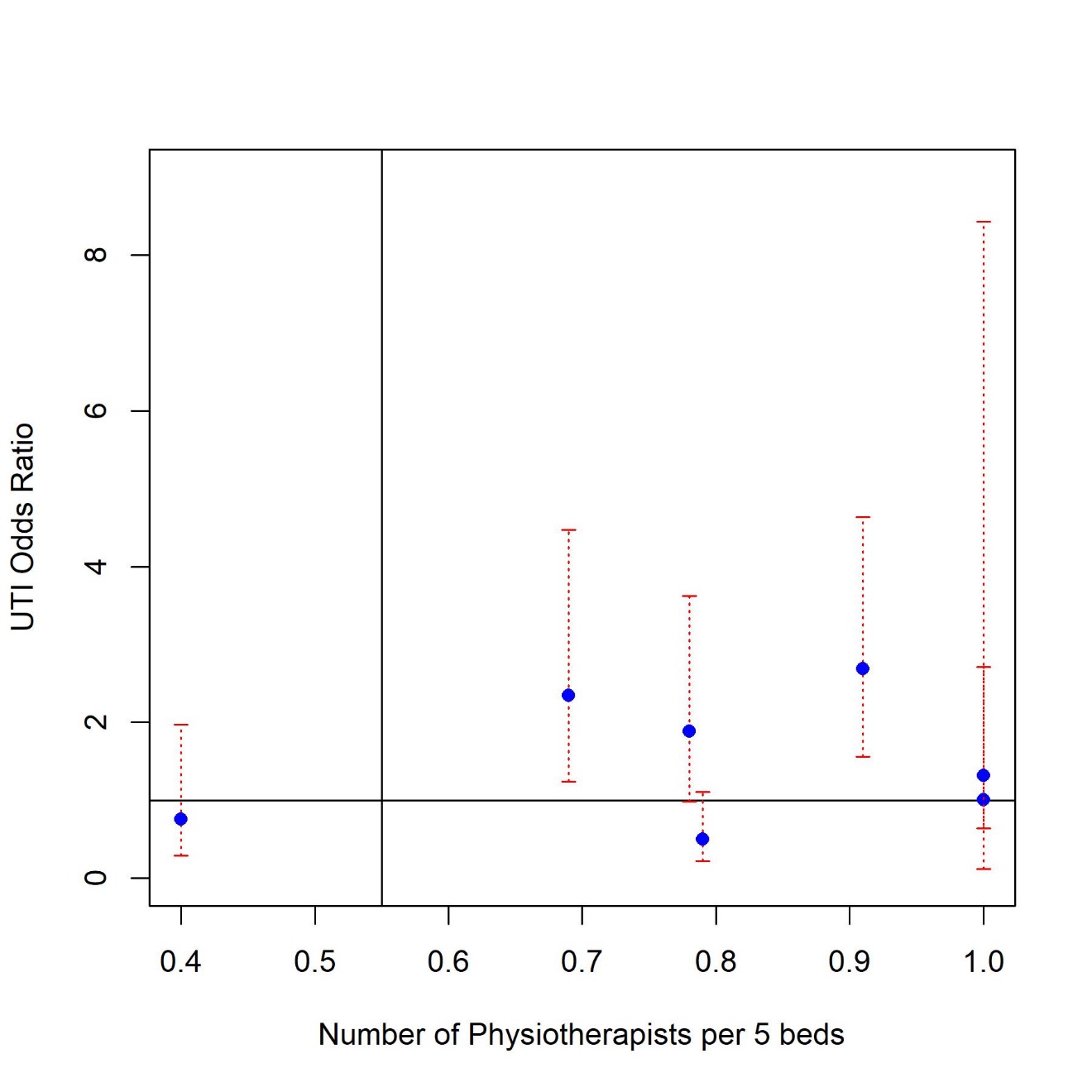


**Supplementary Figure 10.** Model estimates of UTI odds ratio for each hospital against the number of physiotherapists per five stroke unit beds. Horizontal line represents an odds ratio of 1 for reference hospital 1. Vertical line represents the staffing levels of physiotherapists in our reference hospital. Multivariable regression model was adjusted for age, sex, ITU admission, pre-stroke mRS, diabetes mellitus, TACS and pneumonia after multiple imputation for missing covariate data.


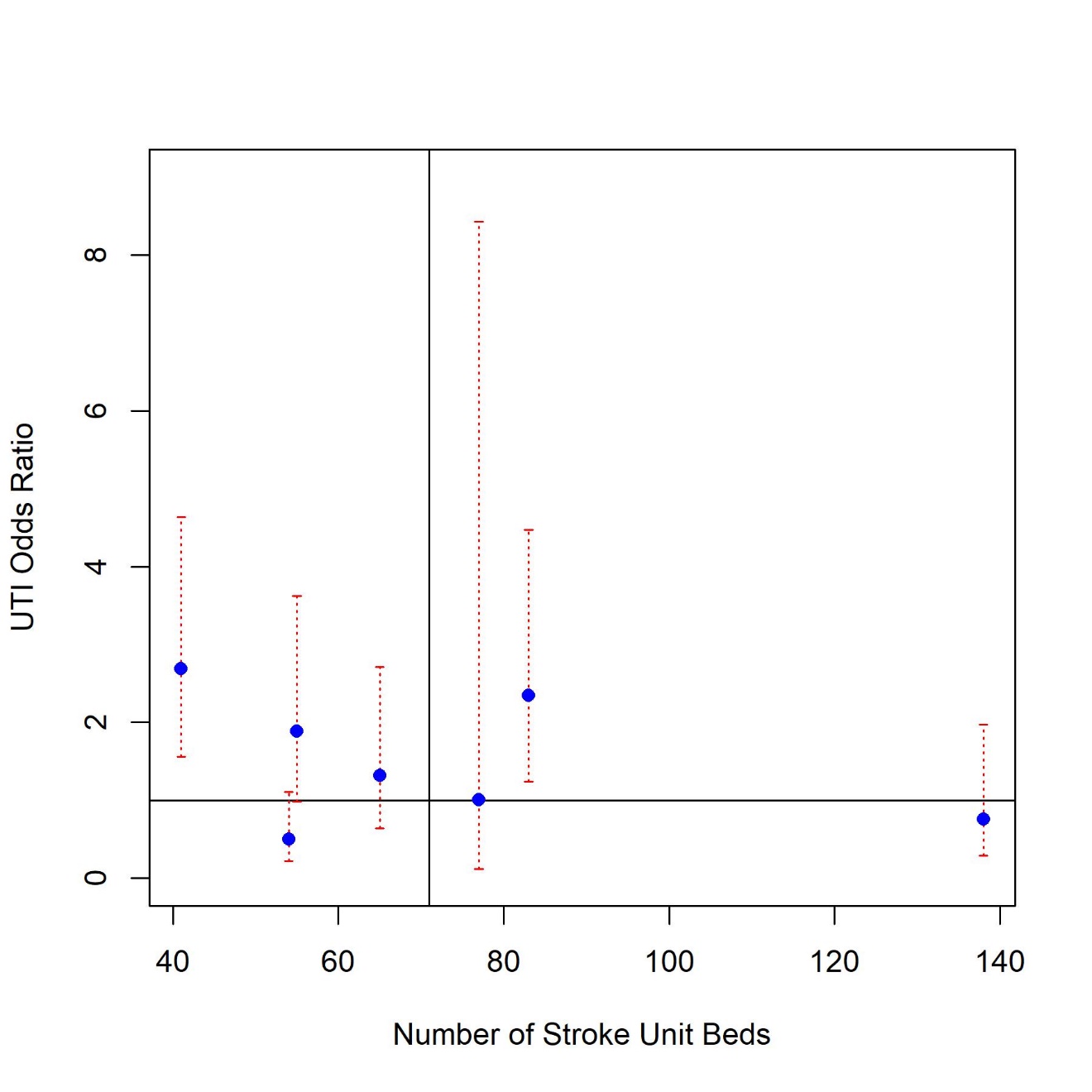


**Supplementary Figure 11.** Model estimates of UTI odds ratio for each hospital against number of stroke unit beds per 100 admissions. Horizontal line represents an odds ratio of 1 for reference hospital 1. Verical line represents the number of stroke unit beds for our reference hospital. Multivariable regression model was adjusted for age, sex, ITU admission, pre-stroke mRS, diabetes mellitus, TACS and pneumonia after multiple imputation for missing covariate data.


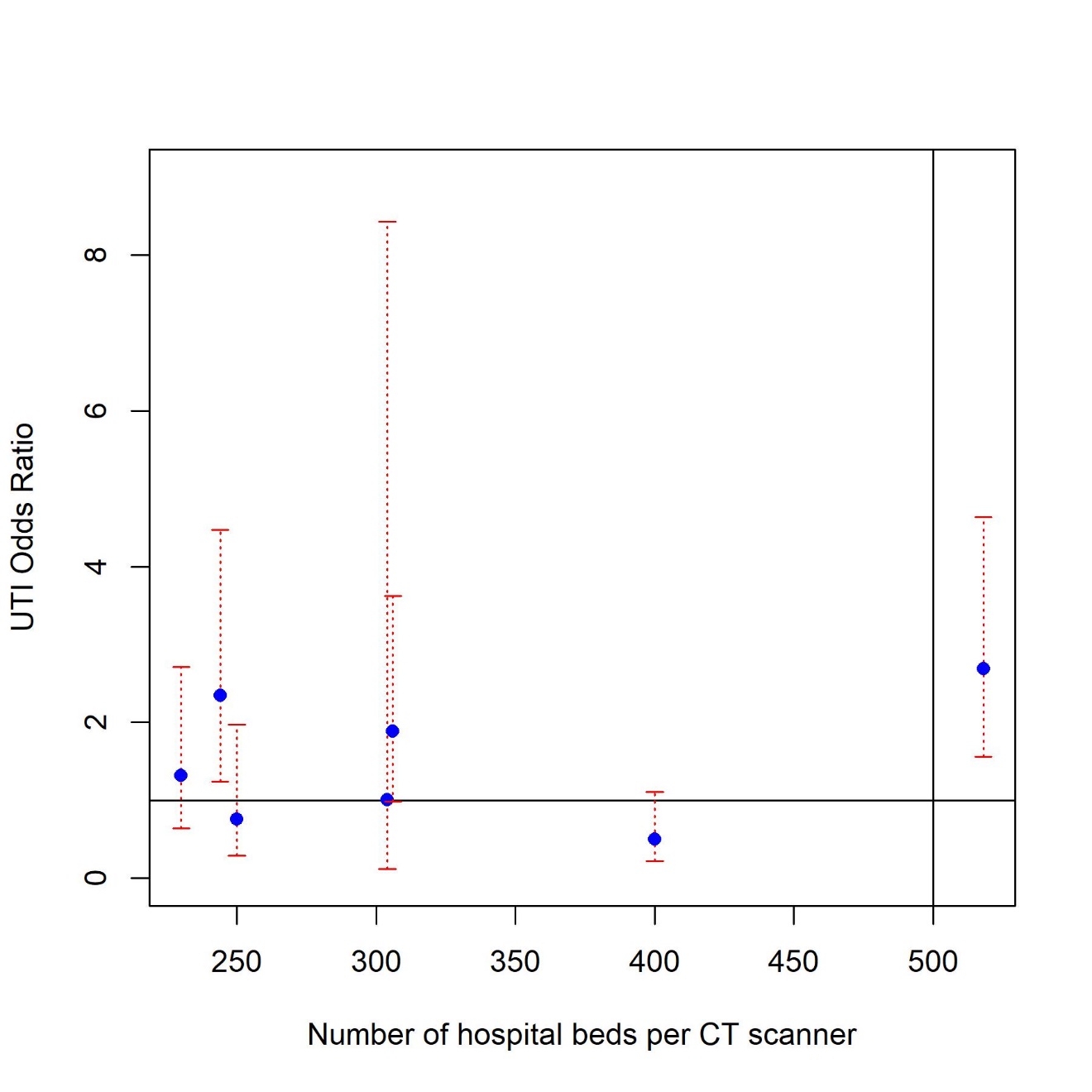


**Supplementary Figure 12.** Model estimates of UTI odds ratio for each hospital against number of hospital beds per CT scanner. Horizontal line represents an odds ratio of 1 for reference hospital 1. Vertical line represents the numer of hospital beds per CT scanner in our reference hospital. Multivariable regression model was adjusted for age, sex, ITU admission, pre-stroke mRS, diabetes mellitus, TACS and pneumonia after multiple imputation for missing covariate data.
